# The Patient Activation Measure-13 (PAM-13) in an oncology patient population: psychometric properties and dimensionality evaluation

**DOI:** 10.1186/s12955-024-02255-w

**Published:** 2024-05-20

**Authors:** Inka Roesel, Daniela Froehlich, Stefanie Joos, Jan Valentini, Holger Mauch, Peter Martus

**Affiliations:** 1 Institute for General Practice and Interprofessional Care, Faculty of Medicine Tuebingen, University Hospital, Tuebingen, Germany; 2 Institute for Clinical Epidemiology and Applied Biostatistics, University Hospital of Tuebingen, Tuebingen, Germany

**Keywords:** Patient activation measure, PAM-13, Confirmatory factor analysis, Exploratory factor analysis, Psychometric evaluation, Item response theory, Partial credit model, Cancer care

## Abstract

**Background:**

Accurate assessment and enhancement of health-related skills among oncology patients are pivotal for optimizing cancer care. The Patient Activation Measure (PAM-13), a questionnaire designed to reflect an individual’s knowledge, skills, and confidence in self-healthcare management, has been validated across diverse countries and settings. Concerns have been raised regarding the cross-situational applicability, as patients with specific diseases and cultural backgrounds interpret questionnaire items differently. This study aimed to examine the structural validity and psychometric properties of the PAM-13 in an oncological patient cohort.

**Methods:**

Baseline data from a longitudinal non-randomized controlled study involving cancer out-patients (*n* = 1,125) from Comprehensive Cancer Centres in Southern Germany were analysed. The German version of the PAM-13 was employed. With classical test and item response theory methods data quality, reliability, convergent and structural validity, as well as psychometric properties were assessed. Exploratory (EFA) and confirmatory factor analyses (CFA) were employed to investigate the postulated unidimensionality of the underlying construct. With a partial credit model (PCM) we examined item fit, targeting, local independence and differential item functioning.

**Results:**

Participants were predominantly female (73.0%) with a breast cancer diagnosis (41.3%). While items were generally well-accepted, ceiling effects were observed and a high mean PAM-13 score (69.7, SD = 14.2) was noted, potentially compromising responsiveness to interventions. Reliability was adequate (Cronbach’s α = 0.81), person and item separation reliability were good to excellent (0.81 and 0.99, respectively). Explorations of the unidimensionality of the construct (EFA, CFA, PCM) yielded inconclusive results, hinting towards a two-factor solution. Item difficulty rankings deviated from the original. No differential item functioning was identified, and local independence was confirmed.

**Conclusions:**

While the PAM-13 serves as a valuable instrument for comprehending and promoting health-related skills in cancer patients, the identification of ceiling effects, disordered item-difficulty rankings, and inconclusive findings regarding unidimensionality contribute to the expanding body of evidence, emphasizing the dependency of PAM-13’s validity and reliability on distinctive characteristics within the population under investigation. Future research should prioritize refining or adding PAM-13 items to better capture the specific health-related challenges within diverse populations, paving the way for more effective patient engagement strategies in oncology.

**Trial registration number:**

DRKS00021779

**Supplementary Information:**

The online version contains supplementary material available at 10.1186/s12955-024-02255-w.

## Introduction

Cancer is a major burden to the affected individuals and a challenge for healthcare systems [1, 2]. With the incidence of cancer considerably rising with age, 29 million annual cases are expected by 2040 based on the projected ageing and growth of most populations around the world [3]. This surge in cancer diagnoses will result in an increased demand for primary health care, as early disease detection and increasingly effective treatments are extending the life expectancy of oncology patients, resulting in more individuals requiring continuous care and management of the long-term sequelae of their illness [4].

While biomedical advancements in treatment regimens are crucial, they alone are not sufficient to meet the needs of cancer patients and their families. High-quality cancer care demands patient-centred communication and individually tailored holistic approaches which address the patients’ preferences and foster the capability to self-manage their acute disease and longer-term follow-up care [5, 6]. This requires an ongoing collaborative relationship between patients and healthcare professionals providing education and resources to empower the individual to take an active role in their healthcare [7]. Research suggests that those who possess the skills and confidence to monitor their condition, to adjust their lifestyle based on their disease status and to make complex decisions, are more likely to experience fewer health crises and functional declines [8], show better adherence to treatment prescriptions [9, 10], report a better perceived health [11, 12] and are ultimately associated with lower healthcare utilization [13] and lower costs [14, 15].

This overarching concept of patient engagement in their own healthcare is also referred to as *patient activation*. To quantify patient activation, Hibbard, Stockard [16] developed the Patient Activation Measure (PAM), originally comprising 22 questionnaire items to assess knowledge, skills and confidence for health self-management - critical aspects for successfully coping with any kind of chronic disease. To enhance feasibility and reduce the administrative burden, the questionnaire was subsequently reduced to 13 items (PAM-13) using Rasch modelling [17]. The PAM-13, as proposed by Hibbard and colleagues, is a unidimensional, Guttman-like scale with items hierarchically ordered by increasing ‘requirement’ of activation, so-called item difficulty. By calculating a summary score derived from the scoring of the individual items (4-point Likert scale, *strongly disagree* to *strongly agree*), patients can be categorized into four increasing levels of activation. These activation levels may assist clinicians and practitioners in providing targeted, individualized patient care and tailored support [17].

### Patient-activation measure (PAM-13): related work

Since the development of the original PAM-13 in 2005, the questionnaire has been translated and validated in various countries, including Germany [18, 19], Norway [20], Italy [21], Singapore [22], Denmark [23], Hungary [24] and many others [25–34]. Participants were typically recruited via convenience sampling from the general population with a heterogenous variety of chronic conditions (e.g., diabetes mellitus, hypertension, rheumatoid arthritis). In other studies, specific disease populations and settings were investigated [35–38]. While the postulated unidimensionality of the PAM-13 was supported by a larger body of research, the one-factor structure could not always be confirmed [20, 28, 29, 32, 33, 39, 40]. In a Norwegian study, a two-factor structure provided a better fit to the data from out-patients awaiting mental health treatment [20]. Among individuals presenting for elective lumbar spine surgery, a three-factor model yielded the best results according to a confirmatory factor analysis [39], whereas Zeng, Jiang [40] showed that a four-factor model, according to the four activation levels, was best suited to explain the variability in the data among persons with diabetes and/or hypertension. These discrepancies may reflect that differences in the diseases studied (chronic or acute, somatic or mental disorders) and also in the cultural background of the sample can result in different factor structures of the PAM-13 [20]. Moreover, in many studies, the items exhibited large ceiling effects [18–20, 22, 34, 41] with only scarce usage of the lowest response level *strongly disagree*, which may result in potential failure to detect changes in activation over time, especially among subjects with already fairly high activation. Furthermore, item separation was found to be low between some items, and the difficulty ranking of items appears to be inconsistent across various study populations and differed from the original PAM-13 [30]. This suggests that the PAM-13 may not be equally generalizable to all populations, as different patient groups find it easier or more difficult to respond affirmatively to certain PAM-13 statements when compared to the original U.S.-American population the questionnaire development was based on [22].

### Research aim

To the best of our knowledge, the underlying structure of the PAM-13 has not been previously investigated specifically in a large oncology patient population encompassing a broader spectrum of different cancer types. The objective of our study was to assess the psychometric properties and construct validity of the PAM-13 in cancer out-patients from Comprehensive Cancer Centres (CCC) in Southern Germany by applying confirmatory and exploratory techniques from classical test theory (CTT) and item response theory (IRT). Healthcare providers and practitioners may benefit from our findings on whether the PAM-13 is a suitable tool for evaluating a patient’s self-management skills in cancer care and for developing tailored intervention programs based on the patient’s activation level.

## Methods and materials

### Study design and eligibility of participants

Data for this analysis were taken from baseline survey information collected for a controlled, non-randomized two-arm (control (CO), intervention group (IG)) longitudinal implementation trial (CCC-Integrativ) of an interprofessional evidence-based counselling program for complementary and integrative healthcare (CIH) in oncology patients [42]. Participants were recruited at four university hospital Comprehensive Cancer Centres (CCC) (Freiburg, Heidelberg, Tuebingen-Stuttgart, Ulm) in the federal state Baden-Wuerttemberg, Germany.

To be eligible for the study, participants had to: [1] be at least 18 years old [2], have a diagnosis of cancer including progression or recurrence within the last 6 months (all cancer types possible) [3], be able to attend counselling on site [4], have treatment at one of the participating CCCs or present themselves there for a second opinion [5], have the need for CIH counselling (IG). Exclusion criteria were language or cognitive impairments preventing patients from completing the survey independently. Eligible participants were recruited using targeted convenience sampling (flyers, newspaper, invitation from treating physicians). For further details on the design and recruitment procedure refer to the study protocol [42].

The study was conducted in accordance with the Helsinki Declaration and has been approved by the Institutional Ethical Committee of the University of Tuebingen, No. 658/2019BO1. All participants gave written informed consent for participation.

### Measurement tools and survey items

All outcomes were self-reported by the patients in questionnaires, except for relevant clinical information extracted from routine medical documentation.

#### Patient activation measure 13

The Patient Activation Measure 13 (PAM-13) is a measure that assesses patient knowledge, skills, and confidence for disease self-management. It is a non-disease-specific tool and can be used across different patient populations. The PAM-13 consists of 13 items on a 4-point Likert scale (1 = *strongly disagree*, 2 = *disagre*e, 3 = *agree*, 4 = *strongly agree*). Item scores are summed up to a raw sum score resulting in theoretical values between 13 and 52, which are then transformed to a standardized metric ranging from 0 to 100. Higher scores indicate a greater patient activation. PAM-13 scores can then be categorized into four hierarchical stages of activation, corresponding to the difficulty of the PAM-13 items: level 1 (patients believe active role is important; items 1–2), level 2 (patients have confidence and knowledge to take action; items 3–8), level 3 (taking action; items 9–11) and level 4 (staying on course under stress; items 12–13). Level categories are formed according to previously defined cut-off thresholds (level 1, ≤ 47; level 2, 47.1–55.1; level 3, 55.2–67; level 4 ≥ 67.1) [43].

For the present analysis, the German version of the PAM-13 (PAM-13-D) was used and scored according to the suggestion by Brenk-Franz, Hibbard [18] (see **Supplement 1**) to only include questionnaires with answers to at least seven items. In case of missing data, the total score was divided by the number of completed items and multiplied by 13 to get the sum raw score. Against the recommendation of the PAM-13 licence owners [44] to remove questionnaires of respondents answering all 13 items with “*strongly disagree*” or “*strongly agree*” as it is suspected that they are not paying attention or are not responding in a truthful way, we refrained from deleting the respective datasets and regarded them as plausible and thus valid answers.

#### Secondary outcome measures

To examine the convergent validity of the PAM-13, correlations with self-efficacy and health-related quality of life were calculated.

##### ***Self-efficacy Scale (SES6G)***

Self-efficacy is a prerequisite of effective self-management in chronic diseases. The SES6G consists of six items with a 10-step Likert scale ranging from 1 ‘*not at all confident*’ to 10 ‘*totally confident*’. The scale is interpreted by calculating a mean score over at least four of the six items, thus allowing a maximum of two missing item responses. Means range from 1 to 10 with higher values indicating higher self-efficacy. The SES6G has a good construct validity and high internal consistency with a reported Cronbach’s α of 0.93 [45].

##### ***Quality of life (EQ-5D-3L)***

The EuroQol five-dimension (EQ-5D-3L) is a valid, generic health-related quality of life (HRQoL) instrument which is self-administered and available in numerous language versions. The EQ-5D consists of two parts: the 20 cm visual analogue scale (EQ-5D VAS), which is rated with scores ranging from 0 (worst) to 100 (best health), and the EQ-5D self-classifier that captures five dimensions of HRQoL, each represented by one item: mobility, self-care, usual activities, pain/discomfort and anxiety/depression. The EQ-5D-3L uses a three-level response option (1 = *no problem*, 2 = *some problems*, 3 = *severe problems*) for each dimension resulting in 5-digit codes that represent the health state of a person. These health states can be converted into an overall index score using population/country-specific weights. Index score ranges differ across weights with higher values representing better health [46].

#### Patient characteristics

We furthermore recorded basic socio-demographic variables, such as age, sex, educational level, body mass index (BMI) and anamnesis data on cancer type, state of diagnosis (first diagnosis, progress, recurrence), treatment intention (curative, palliative, unsure) and metastases (yes, no).

### Sample size

As this study is based on a longitudinal controlled multi-centre trial [42], no separate sample size calculation was conducted for the present analysis. Previous research has demonstrated that for polytomous items, a sample size of at least 250 subjects is required for robust estimates of item parameters and Rasch analysis [22]. A rule of thumb suggests that for exploratory factor analyses a minimum of *N* = 300 participants is required [47, 48]. With a sample size of *N* = 1125 we exceeded this minimum requirement. Stevens [49] posited that the number of participants per variable is a more appropriate way to determine sample size. A person-to-item ratio of at least 10:1 should be ensured [50]. With a person-to-item ratio of 86:1 we have met this prerequisite in our study.

### Data analysis

Data analyses presented here adhered to the Consensus-based Standards for the Selection of Health Measurement Instruments (COSMIN) best practice guidelines for patient-reported outcome measures [51, 52]. We applied methods from both classical test theory (CCT) and item-response theory (IRT) to ensure comparability to previous PAM-13 validation studies [18, 19, 24, 40] and to explicitly model the relationship between an individual’s trait level and their likelihood of providing particular responses to specific items [53, 54].

#### ***Descriptives and data quality of the PAM-13***

The PAM-13 was assessed at item level via mean, median, SD, skewness, kurtosis, percentage of missing data, and extent of ceiling and floor effects. Floor and ceiling effects between 1 and 15% were defined as optimal [55].

#### Classical test theory

##### ***Reliability***

Based on the postulated unidimensionality of the scale, we assessed *internal consistency* with Cronbach’s α [56]. A range of α = 0.7–0.95 was considered adequate [57]. As a violation of the assumption of tau-equivalence can lead to underestimation of the true reliability of a scale [58, 59], we also reported total omega (ω_t_), also known as McDonald’s omega, as recommended by Trizano-Hermosilla and Alvarado [60] in case of approximately normally distributed overall test scores. McDonald’s omega values exceeding 0.7 and 0.8 can be interpreted as demonstrating acceptable and good internal consistency, respectively [61].

Furthermore, *inter-item* and *item-rest correlations* were calculated. Item-rest correlations are the correlations between an item and the scale formed by all other items. High item-rest correlations result in higher α-values and minimally required values for item-rest correlations depend on the scientific background of the study. Rules of thumb state values of > 0.20 to > 0.40 [62]. In a multiple item scale, items should be moderately correlated with each other [27, 63]. Low-correlated items may be too disparate, failing to measure the same construct or idea very well, whereas highly correlated items tend to be too repetitive and are thus redundant [64]. Correlation values of > 0.30 are considered moderate, > 0.50 as strong in this context [65]. Clark and Watson [65] proposed that the average inter-item correlation should fall within the range of 0.15 to 0.50.

##### Convergent validity

Convergent validity was assessed by correlating the PAM-13 scores with the German version of the Self-Efficacy for Managing Chronic Disease 6-Item Scale (SES6G) based on the assumption that the two constructs measure advanced knowledge and coping abilities and are thus conceptually related [66]. Previous research suggested a positive relationship between higher PAM-13 scores and increased self-efficacy [38]. Given that self-efficacy constitutes a part of patient activation, we anticipated a moderate to strong positive correlation between PAM-13 and SES6G. Furthermore, we correlated the PAM-13 with health-related quality of life (EQ-5D), as we conjectured that patients with a higher activation also have a higher quality of life as seen in previous research [67, 68]. We expected a moderate positive correlation with the EQ-5D. Pearson’s product moment correlation was applied for PAM-13 scores and SES6G, Spearman correlation between PAM-13 score and EQ-5D scores (left-skewed). Correlations of *r* ≥ .50 were considered as strong, *r* ≥ .30 as moderate, and *r* ≥ .10 as weak [69].

##### Structural validity

With respect to construct validity, we examined *structural validity*, i.e., the degree to which the scores of an instrument adequately reflect the dimensionality of the construct to be measured [70]. Structural validity was assessed via *confirmatory factor analyses (CFA)* using the R package *lavaan* [71]. The Kaiser-Meyer-Olkin (KMO) statistic for the adequacy of sampling was checked and Bartlett test for sphericity for adequacy of our data for factor analysis. A KMO criterion of greater than 0.5 was regarded as the necessary minimum and 0.8 or higher as optimal for factor analysis. Model fit was assessed by the Root Mean Square Error of Approximation (RMSEA), the Tucker-Lewis index (TLI) and the comparative fit index (CFI), using cut-off values of ≤ 0.05, 0.9 and 0.9 for good fit, respectively. A RMSEA between 0.05 and 0.08 represents an adequate fit, values greater than 0.09 indicate a poor fit. We furthermore calculated the (adjusted) goodness-of-fit index ((A)GFI), which is the proportion of variance accounted for by the estimated population covariance. The GFI and the AGFI should be > 0.95 and > 0.90, respectively. The Standardized Root Mean Square Residual (SRMS) represents the square root of the difference between the residuals of the sample covariance matrix and the hypothesized model. A value of < 0.08 is desirable. For CFA, we tested several theoretical structures of patient activation as postulated in the literature (see Sect. 2.5).

Furthermore, we performed an *exploratory factor analysis* (EFA) using the maximum likelihood method of extraction with oblique rotation (*oblimin*), as we expect factors to be moderately correlated due to the hierarchy of items. We employed multiple decision rules to determine the number of factors (Kaiser’s eigenvalue > 1 rule [72], scree plot [73], parallel analysis [74], Very simple structure (VSS), Velicer’s minimum average partial (MAP) [75]) [50].

##### ***Missing value handling and sensitivity analyses***

Missing values were deleted pairwise to calculate correlations (pairwise-complete correlation matrices). No missing values were imputed. According to Kline [76] a skewness of absolute values > 3.0 and a kurtosis with absolute values > 10.0 indicate “extreme” non-normality, and corrective action should be taken. None of the PAM-13 items exceeded these values (see Table 2). However, to account for less severe violations of multivariate normality of the Likert-type ordinal PAM-item variables, we used full-information maximum likelihood (FIML) with a robust maximum likelihood estimator (MLR) for CFA. As a sensitivity analysis we furthermore applied the weighted least square mean and variance adjusted (WLSMV) method with the drawback of listwise deletion in case of missing data.

#### Item response theory (IRT)

##### ***Partial credit model***

Originally, the PAM-13 was developed using Rasch analysis [17]. Rasch models are probabilistic models assuming that the probability of a given patient responding affirmatively to an item is a logistic function of the relative distance between the item location parameter (*item difficulty*) and the respondent’s ability (*patient ability*, i.e., the individual patient activation in this case) [77]. Based on the postulated unidimensionality of the patient activation measure we implemented a partial credit model (PCM) [78] for polytomous items in accordance with other PAM-13 IRT analyses [21, 31, 41, 79] using the *PCM* function with an conditional maximum likelihood (CML) estimation method from the R package *eRm* [80]. In contrast to the Rasch Rating-scale model, which can also be applied to polytomous items, the PCM has item-specific thresholds (= boundaries between the level categories of an item). We assessed the category probability curves (CPC) [81] to see whether the category calibration increased in an orderly manner: The midpoint where two adjacent curves overlap depicts the threshold, the point of equal likelihood of choosing either response category. As disordered thresholds occurred for item 1, we collapsed the categories “strongly disagree” and “disagree” into one response category and reran the analysis.

Estimated location parameters were calculated, with higher location parameters indicating a greater difficulty of agreeing with the item. Separation distances between adjacent items should be > 0.15 logits, less may indicate redundancy [82]. Guidelines recommend that thresholds should increase by at least 1.4 logits to show sufficient distinction between categories, but no more than 5 logits [83]. Item fit mean square (MNSQ) statistics (infit, outfit) were computed to verify whether the items fitted the expected model. Infit is more sensitive to irregular response patterns according to the person’s ability level, whereas outfit informs about the degree of the item fit [19]. Infit and outfit MNSQ close to 1 indicate a good fit to the model. Values should be between 0.7 and 1.3 on the logit scale [84], lower values indicate possible redundancy, higher values suggest that items might measure something different to the overall scale. The infit and outfit mean squares can be converted to an approximately normalised t-statistic using the Wilson-Hilferty transformation. Values outside the range of (-2, 2) are identified as a potential misfit, indicating either overfitting (< 2) or underfitting (> 2). However, t-statistics have to be interpreted with caution, as they are sensitive to sample size [84].

##### Model reliability

The Person Separation Reliability (PSR) assesses the proportion of observed variance of person ability measures that is not due to error and reflects the ability to differentiate between person’s with different levels of the underlying trait [85, 86]. The concept is related to Cronbach’s alpha but uses the estimates in logits rather than the raw values [87]. An analogous concept for items, the Item Separation Reliability (ISR), reflects how well the items are separated by the persons answering the questionnaire [86]. Values above 0.7 are considered acceptable for PSR and ISR [88].

A person-item map was provided as a graphical representation to display the alignment between the person abilities and item difficulties, so-called “targeting”.

##### Local independence

After conditioning out the effect of the underlying latent factor the questionnaire is measuring, i.e. patient activation, items should not be correlated [89]. The local item independence (LID) assumption is central to IRT models and can be evaluated by calculation of Yen’s Q_3_ statistics [90, 91], a pairwise correlation index of the residual from the IRT model. A substantial residual correlation could indicate that the response to one item influences the response to another and violations may lead to overestimations of reliability and problems related to construct validity [90].

Christensen, Makransky [90] suggested that that LID should be considered relative to the average observed residual correlation (${\overline Q _3}$) and proposed to use a critical threshold of 0.2 above ${\overline Q _3}$ for the Q_3_ values to detect undesirable local dependence.

##### ***Unidimensionality***

To verify unidimensionality, we performed a Principal Component analysis of (standardized) Residuals (PCAR) which creates potential secondary dimensions (“contrasts”) based on unexplained variance of the residuals [92]. To substantiate the hypothesis that the residuals are random noise and thus support the assumption of unidimensionality, the eigenvalue of the first residual contrast should be less than 3 and the first contrast should account for less than 15% of the variance [93, 94].

##### ***Differential item functioning (Measurement invariance)***

Differential item functioning (DIF) detects item bias in the internal structure. DIF occurs when respondents of different groups have the same ability, but a different probability of success on an item. According to DIF in previous PAM-13 validation studies, we tested for DIF with respect to sex [19, 21–23, 25, 31], age [19, 21, 23, 25], and education [21–23, 79]. Based on differences found in PAM-13 scores on certain health characteristics in our study population, we tested for DIF regarding status of diagnoses (first diagnosis, progression, recurrence) and intervention group (CO, IG). The *lordif* package in R was used [95], which performs ordinal logistic-regression DIF. First, an overall Anderson Likelihood Ratio (LR) test was conducted, which is a global assessment of the null hypothesis that scaling is equal between two groups. For the continuous variable age, the sample was divided into three groups (younger than 44 years, 44 to 64 years, 65 years and older). In case of a statistically significant result of the overall LR-test (*p* < .01, Bonferroni-adjusted for the five grouping variables), a LR chi-squared test was conducted for each item, ‘flagging’ biased items for uniform or non-uniform DIF [96]. It has been suggested that unidimensionality of the scale is supported when no more than 5% of the items exhibit DIF [31, 97, 98].

##### ***Sensitivity analysis***

As a sensitivity analysis for the widely utilized 1-parameter PCM in numerous PAM-13 validation studies, we additionally implemented a generalized partial credit model (GPCM) to relax the assumption of uniform discriminating power across test items [99]. In the GPCM, an additional slope parameter ${\alpha }_{i}$ for each item $i$ is introduced, allowing for differential discrimination ability of the PAM-13 items. As the GPMC is not supported by the *eRm* package that we used for the PCM, we performed the GPCM using the R package *mirt*, developed by Chalmers [100].

All analyses above were performed with R version 4.1.3 and R Studio (version 2022.02.1). A type I error rate of 0.05 was used to determine statistical significance, whenever multiple testing correction was not applicable.

### Theoretical patient activation models for CFA

Three CFA models were considered as competing alternative versions: a one-factor [17], a two-factor [20], and a four-factor model [40].

In the one-factor model, all 13 items of the PAM were specified to constitute one general latent factor. In developing the PAM-13, the unidimensional structure was posited by Hibbard, Mahoney [17]. The measure was constructed using Rasch analysis on data from a telephone survey (*N* = 1,515) with randomly selected adults in the US, aged 45 years and older. 79% of the sample reported at least one chronic disease. Items proved to be well-spaced along the measurement scale from easy (item 1) to difficult (item 13). The one-factor structure was corroborated by psychometric evaluations of the German version of the PAM-13 with an explorative principal component analysis [18, 19] and a Rasch model [19], as well as in other regional validation studies and patient populations.

Moljord, Lara-Cabrera [20] conducted an exploratory factor analysis with 273 out-patients waiting for treatment in community mental health centres. In the resulting two-factor model, items 4–13 (related to “knowledge and self-confidence”) were specified to identify with the first factor, the first three PAM items (related to “believing active role important and responsibility”) were specified to identify with the second factor. This bi-factorial solution explained 48.07% of the variance, the two components revealed a correlation of 0.41.

The four-factor version is based on the four hierarchical activation levels as described in Sect. 2.2.1 above. Zeng, Jiang [40] conducted a confirmatory factor analysis on cross-sectional data from 519 patients with hypertension and/or type 2 diabetes managed at community health centres.

## Results

### Participants: Socio-demography and health characteristics

In the original study, 1128 participants provided data at baseline (IG: 685, CO: 443). Three subjects had to be excluded for our analyses, as they had filled out less than seven PAM-13 items. Finally, we included 1125 subjects into our analyses (IG: 685, CO: 440). Sociodemographic information and health characteristics are displayed in Table 1. The participants’ mean age was 57 years (SD = 12.2, range 18–88), most of them living in a relationship/being married (*n* = 860, 78.3%). The majority was employed full- or part-time (*n* = 624, 56.3%) and over one-third held a university/college degree. A frequent oncological diagnosis was breast cancer (*n* = 465, 41.3%), which aligns with the fact that participants were predominantly female (*n* = 821, 73.0%).

**Table 1:** Patient characteristics, anamnesis at study entry and PAM-13 scores within strata

	TOTAL	CO	IG	*p* ^*^	PAM-13	*p* ^#^
	*N* = 1125	*N* = 440	*N* = 685		Scores	
**Socio-demographics**						
		Mean (SD)			Pearson r	
**Age (** *N* ** = 1125)**	57.1 (12.2)	59.8 (12.3)	55.4 (11.8)	**< 0.001** ^$^	0.014	*p* = .647
**BMI (** *N* ** = 1107)**	24.8 (5.13)	25.3 (5.43)	24.5 (4.89)	**0.012** ^$^	-0.030	*p* = .313
	N (%)				Mean (SD)	
**Sex (** *N* ** = 1125)**				**0.015** ^&^		T = 0.76, *p* = .448
Male	304 (27.0%)	137 (31.1%)	167 (24.4%)		69.1 (15.1)	
Female	821 (73.0%)	303 (68.9%)	518 (75.6%)		69.9 (13.9)	
**Education (** *N* ** = 1113)**				**< 0.001** ^&^		F = 3.23, *p* = .022
University/College degree	405 (36.4%)	123 (28.4%)	282 (41.5%)		69.8 (13.3)	
Higher education qualification	179 (16.1%)	55 (12.7%)	124 (18.2%)		70.0 (13.6)	
Intermediate secondary school	343 (30.8%)	150 (34.6%)	193 (28.4%)		70.9 (14.5)	
Basic school up to 9 yrs/no qualification	186 (16.7%)	105 (24.2%)	81 (11.9%)		70.0 (15.9)	
**Employment (** *N* ** = 1109)**				**< 0.001** ^&^		F = 2.10, *p* = .099
Full-time	352 (31.8%)	124 (28.8%)	228 (33.6%)		70.7 (13.6)	
Part-time	272 (24.5%)	79 (18.3%)	193 (28.5%)		70.2 (13.4)	
Not employed	96 (8.65%)	43 (9.98%)	53 (7.81%)		66.8 (15.4)	
Retired	389 (35.0%)	185 (42.9%)	204 (30.0%)		69.3 (14.9)	
**Marital status (** *N* ** = 1099)**				0.178^&^		T = 1.43, *p* = .154
Single	239 (21.7%)	103 (24.0%)	136 (20.3%)		68.4 (15.4)	
In a relationship/married	860 (78.3%)	327 (76.0%)	533 (79.7%)		70.0 (13.9)	
**Anamnesis**						
**Main Diagnosis** ^**$**^ **(** *N* ** = 1125)**				**0.036** ^&^		F = 0.23, *p* = .878
Mamma	465 (41.3%)	165 (37.5%)	300 (43.8%)		70.0 (14.0)	
Digestive organs	232 (20.6%)	102 (23.2%)	130 (19.0%)		69.2 (14.4)	
Female genitals	118 (10.5%)	56 (12.7%)	62 (9.0%)		70.2 (14.1)	
Other	310 (27.6%)	117 (26.6%)	193 (28.2%)		69.4 (14.4)	
**Treatment intention (** *N* ** = 1125)**				**0.001** ^&^		F = 2.13, *p* = .120
Curative	538 (47.8%)	192 (43.6%)	346 (50.5%)		70.6 (13.5)	
Palliative	406 (36.1%)	156 (35.5%)	250 (36.5%)		68.9 (14.3)	
Unsure	181 (16.1%)	92 (20.9%)	89 (13.0%)		68.7 (15.7)	
**Diagnosis Status (** *N* ** = 1125)**				0.410		F = 4.04, *p* = .018
First Diagnosis	680 (60.4%)	271 (61.6%)	409 (59.7%)		70.6 (14.4)	
Progress	331 (29.4%)	131 (29.8%)	200 (29.2%)		67.9 (14.0)	
Recurrence	114 (10.1%)	38 (8.64%)	76 (11.1%)		69.5 (13.5)	
**Metastases (** *N* ** = 1048)**				0.205		T = 1.91, *p* = .056
No	577 (55.1%)	218 (52.5%)	3659 (56.7%)		70.5 (14.4)	
Yes	471 (44.9%)	197 (47.5%)	274 (43.3%)		68.8 (15.2)	

* Between-group (CO vs. IG) differences, $ = Student’s t-test, & = Chi-squared, T = Student’s t-test, F = ANOVA test; # = p values for associations between patient characteristics and PAM-13 scores or between-group differences are unadjusted for multiple testing; $ = Only the main primary diagnosis at study entry is presented here

### Data quality PAM-13 items and scores

**Fig. 1:**
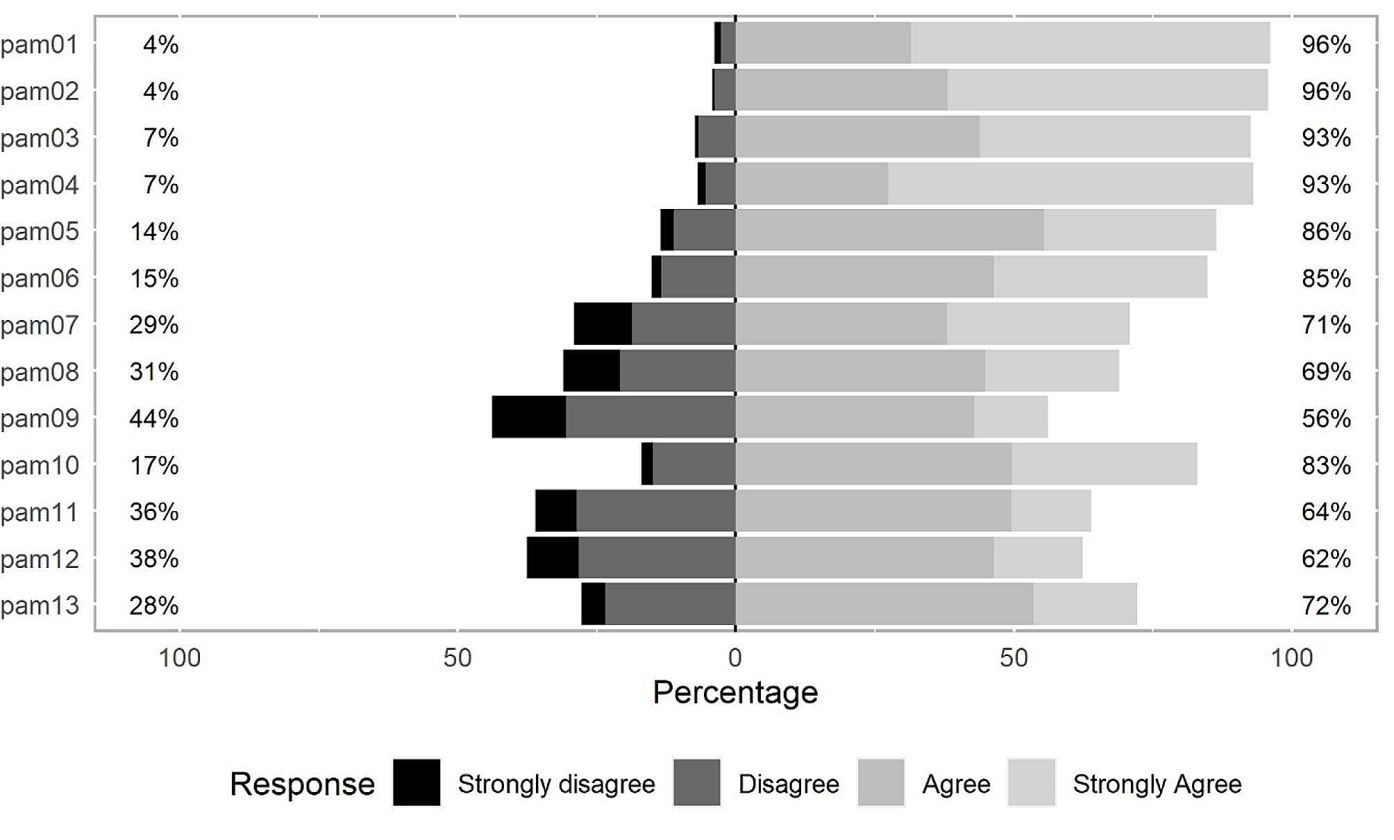
PAM-13 item responses (excluding missing data); *N* = 1125

Descriptives of the 13 PAM-items are displayed in Table 2. Overall, 85.2% (*N* = 959) of the study participants provided complete questionnaires, only 1.54% (*N* = 225) of all PAM-13 values were missing. In general, item-missingness was low with percentages ranging between 0% (item 5) to 1.7% (item 9) with the exception of item 4 (“*I know what each of my prescribed medications does*”) with 9.2% missingness. All 13 items met the standards of a small floor effect (range 0.45–13.38%). Regarding ceiling effects, all items except for items 9 (13.20%) and 11 (14.40%) exhibited a ceiling effect larger than the threshold of 15% (range: 16.06–65.72%) with the tendency of lower PAM-13 items having larger ceiling effects. None of the patients responded with *strongly disagree* to all PAM items, 16 (0.14%) subjects answered *strongly agree* to all thirteen questions. Figure 1 depicts the selected categories (*strongly disagree*, *disagree*, *agree*, *strongly agree*).

The overall mean PAM-13 score was 69.68 (SD = 14.21) with a range of 17.9 to 100 and a slightly left-skewed distribution (skewness=-0.24, kurtosis = 0.06). The PAM-13 mean scores in the control and intervention group exhibited no significant difference (CO: mean = 68.98 (SD = 14.95), IG: mean = 70.13 (SD = 13.7), *p* = .191). Regarding the associations between participant characteristics and patient activation, no significant correlations or differences were found in the mean PAM-13 scores except for educational level and diagnosis status (Table 1). Pairwise post-hoc tests (Bonferroni-adjusted) revealed a significant difference in PAM-13 scores between no qualification/basic school education and a secondary school education (*p* = .013) and between first diagnosis and progress (*p* = .014).

**Table 2:** Data quality and description of the PAM-13 items

Item	*N* = 1125	Missings *N*, %	mean	sd	median	min	max	skew	kurtosis	se	Floor (%)	Ceiling (%)	Item-rest correlation
1	1115	10 (0.9)	3.60	0.60	4	1	4	-1.53	2.71	0.02	1.17	**64.57**	0.373
2	1112	13 (1.2)	3.53	0.59	4	1	4	-0.98	0.63	0.02	0.45	**57.73**	0.424
3	1115	10 (0.9)	3.41	0.64	3	1	4	-0.77	0.19	0.02	0.63	**48.79**	0.415
4	1021	104 (9.2)	3.57	0.66	4	1	4	-1.56	2.20	0.02	1.37	**65.72**	0.362
5	1125	0 (0.0)	3.15	0.70	3	1	4	-0.62	0.53	0.02	2.31	**31.02**	0.483
6	1124	1 (0.1)	3.22	0.73	3	1	4	-0.62	-0.09	0.02	1.69	**38.43**	0.369
7	1113	12 (1.1)	2.93	0.97	3	1	4	-0.56	-0.66	0.03	10.51	**32.88**	0.488
8	1111	14 (1.2)	2.83	0.91	3	1	4	-0.47	-0.54	0.03	10.26	**24.03**	0.472
9	1106	19 (1.7)	2.56	0.88	3	1	4	-0.19	-0.68	0.03	13.38	13.20	0.494
10	1118	7 (0.6)	3.14	0.74	3	1	4	-0.54	-0.08	0.02	2.06	**33.36**	0.456
11	1118	7 (0.6)	2.71	0.80	3	1	4	-0.29	-0.32	0.02	7.42	14.40	0.517^a^
12	1108	17 (1.5)	2.69	0.85	3	1	4	-0.28	-0.50	0.03	9.30	**16.06**	0.586^a^
13	1114	11 (1.0)	2.87	0.76	3	1	4	-0.36	-0.10	0.02	4.31	**18.67**	0.488

sd = standard deviation; se = standard error; for ceiling effects values above 15 are bold; ^a^ = item-rest correlation > 0.5

### CTT-based analyses

#### Reliability

Cronbach’s alpha was 0.81 (95%-CI: 0.80–0.83), indicating an adequate internal consistency. Omega (ω_t_) was found to be 0.84. The minimum individual inter-item correlation was *r* = .13 (item 3 and 4) and did not exceed *r* = .54 (item 11 and 12). All other inter-item correlations fell into the ideal range of *r* = .15–0.5, with an overall average inter-item correlation of 0.25. Item-rest correlations are displayed in Table 2 and were moderate (items 1–10, 13) to strong (items 11, 12).

#### Convergent validity

The Pearson correlation coefficient of *r* = .39 (*p* < .001, *n* = 1113) revealed a moderate correlation between the mean sum scores of the PAM-13 and the SES6G. The Spearman correlation coefficient amounted to *r* = .22 between PAM-13 and EQ-5D scores (*p* < .001, *n* = 1090).

#### Structural validity: factor analyses

The measure of sampling adequacy showed an adequate correlation of items (KMO criterion = 0.85). Bartlett’s test of sphericity was performed to explore the factorability of the correlation matrix and proved to be adequate, as the null hypothesis could be rejected (χ^2^ [78] = 3166.19, *p* < .001). The correlation matrix used for factor analyses can be found in Supplement 2.

##### ***Confirmatory factor analysis (CFA)***

**Fig. 2:**
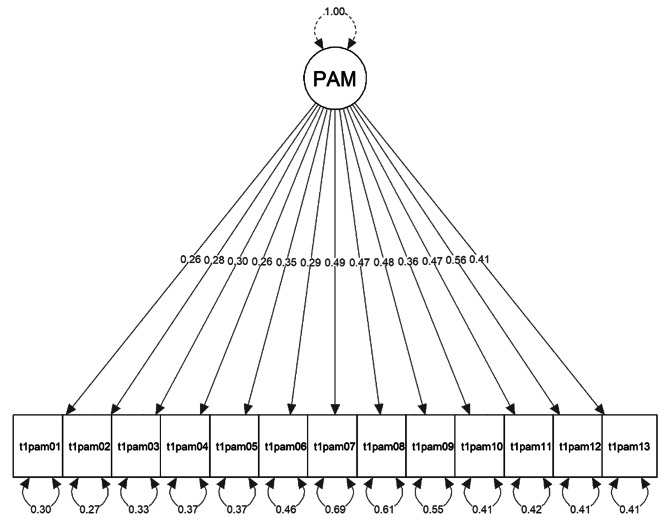
*One-factor model*, robust FIML, variance-standardization method, df = 65, uncorrelated errors

**Fig. 3:**
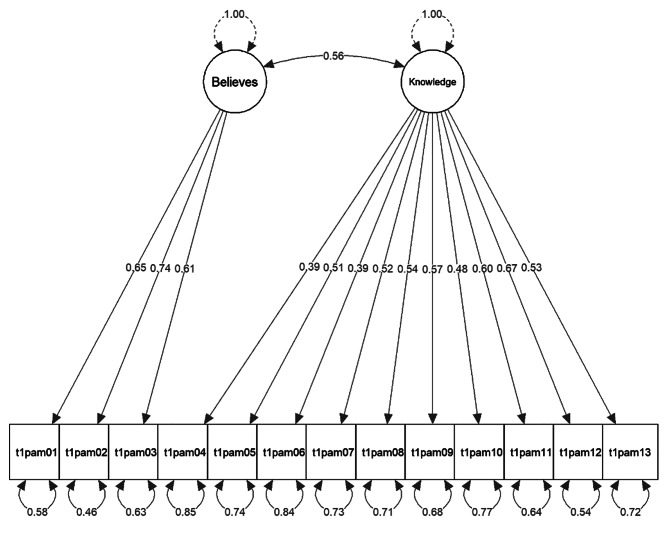
*Two-factor model*, robust FIML, variance-standardization method, df = 64, uncorrelated errors

**Fig. 4:**
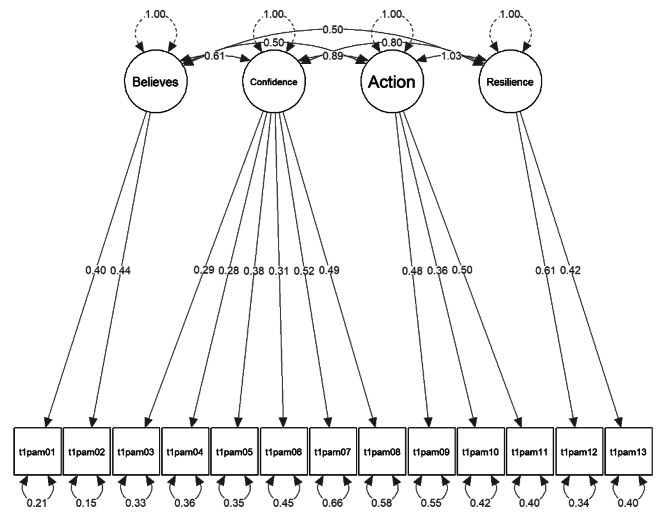
*Four-factor model*, robust FIML, variance-standardization method, df = 78, uncorrelated errors

Model fit indices of the confirmatory factor analysis for the three alternate models are presented in Table 3 and model figures are displayed in Figs. 2, 3 and 4. In all three models, each parameter from the manifested indicators to the latent variables was statistically significant (*p* < .05) and no localized strain was present.

**Table 3:** Fit indices for confirmatory factor analysis models

Model	RMSEA (90% CI)	CFI	TLI	GFI	AGFI	SRMR
Robust FIML (*n* = 1125)						
*1-factor*	0.086 (0.081; 0.092)	0.779	0.737	0.993	0.989	0.063
*2-factor*	0.064 (0.058; 0.070)	0.882	0.856	0.996	0.993	0.046
*4-factor*	0.073 (0.067; 0.080)	0.856	0.815	0.995	0.991	0.053
WLSMV with listwise deletion (*n* = 959)						
*1-factor*	0.075 (0.068; 0.082)	0.840	0.808	0.999	0.998	0.061
*2-factor*	0.053 (0.046; 0.060)	0.922	0.904	0.999	0.999	0.043
*4-factor*	0.069 (0.062; 0.076)	0.877	0.837	0.999	0.999	0.052

RMSEA = Root Mean Square Error of Approximation; CI = Confidence Interval; CFI = Comparative fit index; TLI = Tucker-Lewis index

GFI = Goodness-of-fit; AGFI = Adjusted goodness-of-fit; SRMR = Standardized Root Mean Square Residual

The data did not confirm the single-factor structure of patient activation, nor was the four-factor model based on the four activation stages particularly suited to explain the variability in the data according to the fit statistics. The two-factor model exhibited a reasonable fit and significantly outperformed the one-factor solution (Chi-square difference = 135.36, *p* < .001). With a correlation of *r* = .56 < .8 between the two constructs “Believes” and “Knowledge”, sufficient discriminant validity was given [101]. All factor loadings were ≥ 0.39, with the lowest loadings of item 4 and 6. Sensitivity analyses with WLSMV estimator and listwise deletion generally resulted in slightly better fit indices, however, overall results were consistent with the MLR estimator versions.

##### ***Exploratory factor analysis (EFA)***

Conducting an exploratory factor analysis, the evidence for unidimensionality of the PAM-13 was inconclusive. The VSS complexity of 1 (max = 0.76) as well as MAP (min = 0.03) indicated a one-factor solution, scree plot and parallel analysis suggested one or two factors (refer to **Supplement 3**). According to the Kaiser criterion (eigenvalue (ev) > 1), a three-factor model was proposed (ev_1_ = 4.834, ev_2_ = 1.450, ev_3_ = 1.022), however, this rule is among the least accurate criteria for assessing factor retention [102, 103], and a three- or four-factor model resulted in insufficient primary loadings for several items (low-loading, cross-loading). In the 2-factor solution item clustering was identical to the proposed bi-factorial model tested with CFA: Items 4–13 are identified with factor 1, representing “Knowledge and self-confidence”, items 1–3 are identified with factor 2, reflecting the “Belief that an active role and responsibility is important”. All item loadings were greater than 0.35, exceeding the rule of thumb of minimum loadings of 0.32 suggested by Tabachnick and Fidell [48]. In total, the two factors explained 48.35% of the variance (Factor 1: 37.19%; Factor 2: 11.16%). The two components revealed a correlation of 0.5. Communalities ranged from 0.15 to 0.59; especially for item 4 and 6 communalities were low (0.16 and 0.15, respectively). A follow-up reliability analysis found an omega total of 0.83 for factor 1 and 0.71 for factor 2. Cronbach’s alpha values were lower (see Table 4).

**Table 4:** Factor loadings and communalities based on a two-factor EFA with maximum likelihood extraction method and oblimin rotation (*n* = 1,125)

Item	Loadings		
	Factor 1	Factor 2	Communalities
***1***		0.621	0.40
***2***		0.778	0.59
***3***		0.521	0.34
***4***	0.374		0.16
***5***	0.476		0.26
***6***	0.351		0.15
***7***	0.491		0.27
***8***	0.604		0.32
***9***	0.646		0.36
***10***	0.367		0.23
***11***	0.598		0.37
***12***	0.648		0.45
***13***	0.427		0.27
***Crossloadings > 0.32***	none	none	
***Eigenvalues***	4.835	1.450	
***Variance***	37.19%	11.16%	
***Omega total***	0.83	0.71	
***Cronbach’s alpha***	0.79	0.69	

Note: Factor loadings < 0.2 are suppressed

### IRT-based analyses – partial credit model

All reported findings pertain to analyses where the item categories “strongly disagree” and “disagree” have been combined (see Sect. 2.4.2).

#### ***Reliability***

Item Separation Reliability (ISR = 0.99) was excellent, and Person Separation Reliability (PSR = 0.81) was good, both exceeding the minimal acceptable threshold of 0.7.

#### ***Item statistics***

Item statistics of the partial credit model are presented in Table 5. Item 4 (“I know what each of my prescribed medications do”) was the only item with a significant chi-square statistic (*p* = .013 after Bonferroni correction) measuring the discrepancy between observed and expected frequencies for each response category. Outfit MNSQ ranged from 0.803 to 1.274 and infit MNSQ from 0.806 to 1.155, indicating an adequate goodness-of-fit. However, absolute t-values exceeded the threshold of 2 for several items (outfit: item 2,4,5,7,12,13; infit: item 2,3,4,5,7,9,11,12,13).

**Table 5:**
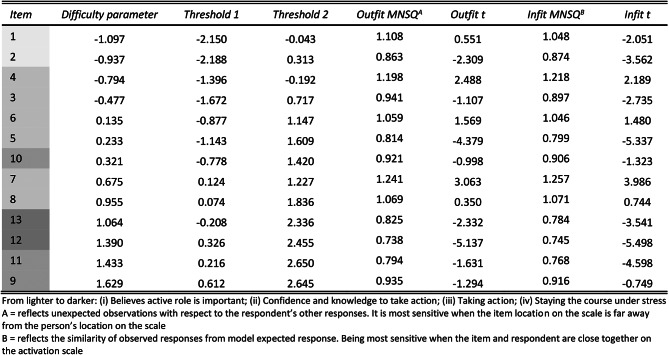
Item fit statistics (*strongly disagree* and *disagree* categories combined)

**Fig. 5:**
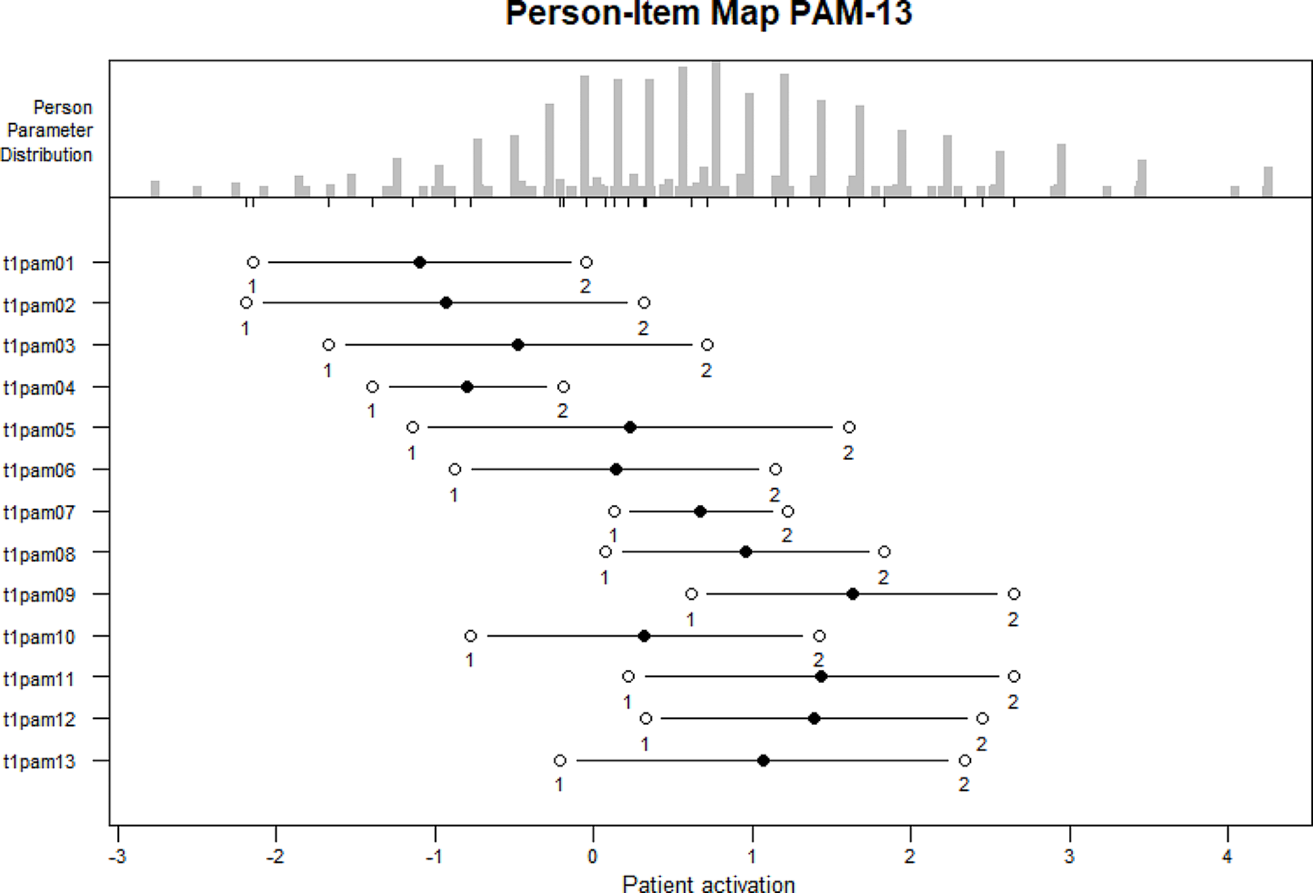
Person-item map for the PAM-13. Black dots: Location (difficulty) parameters; White dots: Category thresholds (*strongly disagree* and *disagree* categories combined)

The person-item map (Fig. 5) displays the person parameter distribution on the latent underlying dimension (i.e., patient activation) and the item difficulties on a logit scale. The mean item difficulty is set at 0. The mean location for person was 0.83 (SD = 1.26). Black dots represent the location parameters (item difficulty), which ranged from − 1.097 to 1.629 logits (Table 5). After collapsing the categories “strongly disagree” and “agree” all items were well-ordered. The original item difficulty ranking as proposed by Hibbard, Mahoney [17] could not be confirmed. For example, item 10 with a location parameter of 0.321 was less difficult than item 7 with a location parameter of 0.675. Item 9 proved to be the most difficult item with a difficulty parameter of 1.629. Separation difficulties were seen between some adjacent items: Lower separation than 0.15 logits were found between items 5 and 6 (difference = 0.095), items 5 and 10 (difference = 0.088), items 13 and 8 (difference = 0.108) and items 11 and 12 (difference = 0.045). White dots in the person-item map represent the thresholds. Spacing was adequate except for minor problem of distinction between thresholds for item 4 and 7.

#### ***Unidimensionality***

In the results of the PCAR, the eigenvalue of the first contrast was 1.83 and variance explained amounted to 14%, supporting the evidence of unidimensionality. The items with the strongest positive loadings on the first contrast were items 1 (0.49), 2 (0.55) and 3 (0.54). Items with the largest negative loadings were items 8 (-0.49) and 9 (-0.48).

#### ***Local independence***

Yen’s Q3 residual correlation statistic between the items 1 and 2 was 0.203, for all other item pairs the residual correlation did not exceed the 0.2 above average threshold and local independence was given.

#### ***Differential item functioning (DIF)***

None of the overall Anderson LR-tests showed statistically significant differences for the tested subgroups (sex: LR-value: 34.72, df = 25, *p* = .093; age: LR-value: 52.99, df = 50, *p* = .360; education: LR-value: 60.76, df = 75, *p* = .883; diagnosis status: LR-value: 49.64, df = 50, *p* = .488; intervention group: LR-value: 23.87, df = 25, *p* = .527). In line with these results, using the R^2^ change threshold of ≥ 0.02 as a criterion for differential item functioning, in none of the grouping variables (intervention group, age, sex, education, diagnosis status) DIF was present. We therefore considered activation values across groups as robust.

### Comparison of item difficulties across different study populations

In analogy to investigations by Moreno et al. [30] we compared the PAM-13 item difficulty order in our study with item rankings found in the literature for other countries and populations (refer to Table 6). Additionally, Spearman correlations were computed to assess the associations between the item ranks in the specific studies and the original item order derived by the PAM-13 developers [17]. Histograms illustrating the distributions of rankings for each item across the 14 studies can be found in **Supplement 4**. Item ranks for our CCC-Integrativ sample were fairly similar to the original order (*r* = .885). However, there were some deviations, with item 10 (“I am able to maintain the lifestyle changes for my health that I have made”) dropping into 7th position and thus requiring a lower level of activation than initially anticipated. Additionally, item 13 (“I am confident that I can maintain lifestyle changes, like eating right and exercising, even during times of stress”) exhibited a lower difficulty in our sample (10th position). Conversely, item 9 (“I know what treatments are available for my health problems”) was ranked as the most difficult. Across the studies presented in Table 6, the item order in our study demonstrated the strongest correlation with the Italian version of the PAM-13 [21] validated in a population of patients with chronic conditions (*r* = .918). The lowest correlation was observed for the Korean version of the PAM-13, investigated in a sample of patients with osteoarthritis [25].

### Sensitivity analysis

Comparisons of the PCM and GPCM revealed a significant difference between the two models (LR-test, ${{\rm X}}^{2}=58.46, p<.001$). Further model diagnostics, detailed in **Supplement 5**, also favored the GPCM over the PCM. This preference was evidenced by lower values of the Akaike Information Criterion (AIC), Sample-Size Adjusted Bayesian Information Criterion (SABIC), and Root Mean Square Error of Approximation (RSMEA). Additionally, higher values for Tucker-Lewis Index (TLI) and Comparative Fit Index (CFI) indicated a superior model fit for the GPCM. Upon inspecting the item statistics, the outfit Mean Square (MNSQ) ranged from 0.872 to 0.940 and the infit MNSQ from 0.868 to 0.955, suggesting a good fit. However, the outfit z-statistics exceeded the absolute value for several items, which is consistent with the findings for the PCM. The mean location for person was found to be -0.001 (SD = 0.91), compared to 0.83 (SD = 1.26) for the PCM. The order of item difficulty was very similar to that established for the PCM model, except for item 4 moving to position 1, consequently causing items 1 and 2 to move up one position. Item discrimination ranged from 0.787 (item 6) to 1.595 (item 12: “I am confident I can figure out solutions when new situations or problems arise with my health condition”), with a mean value of 1.076 and SD = 0.214. For further details on item fit statistics and graphical representations, refer to Supplement 5.

**Table 6:**
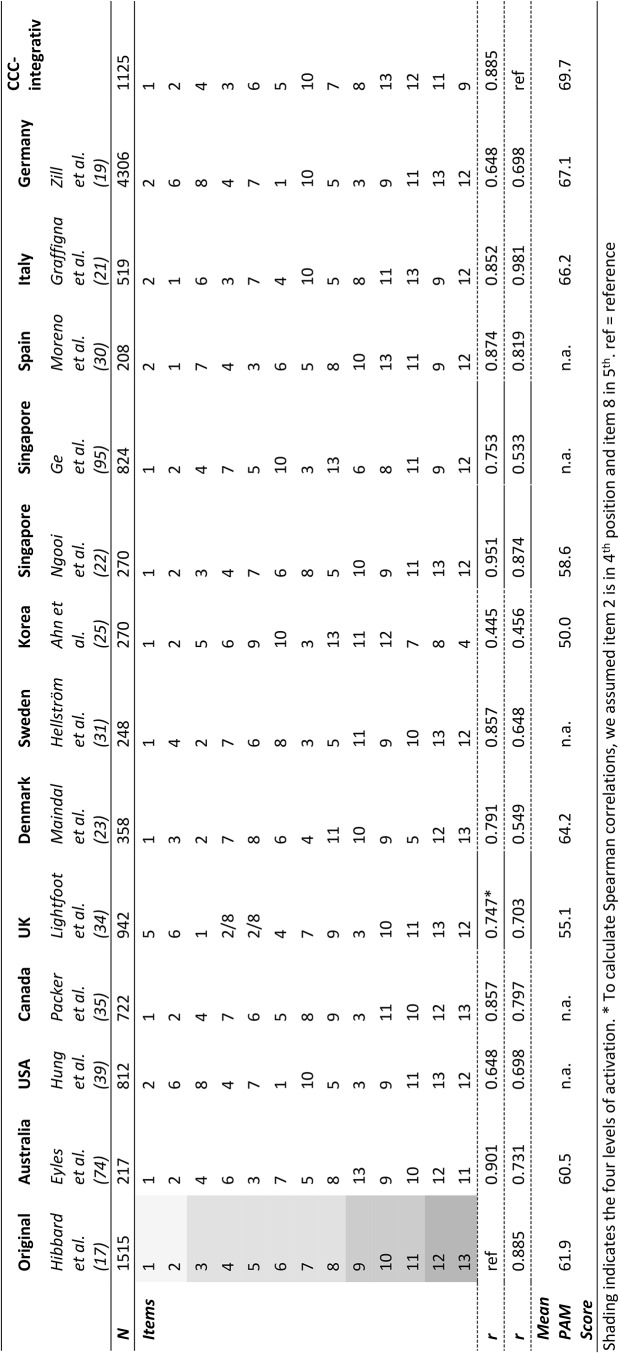
Item difficulty ranks in different populations

## Discussion

The presented study aimed to rigorously investigate the properties of the German PAM-13 among a heterogeneous group of oncology outpatients from CCCs and to scrutinize the postulated underlying unidimensional structure. The overarching goal was to verify the validity and reliability of the PAM-13 as a valuable instrument to measure patient activation within the specific context of cancer care. While the results indicated a reasonable performance of the PAM-13 in certain aspects, the factor structure exhibited ambiguity and conclusive confirmation of unidimensionality could not be reached. The analysis of the psychometric properties also pointed towards potential areas for improvement of the PAM-13, particularly with regard to item levels, order of item difficulties, and the overall range of the scale.

Generally, the PAM-13 items were well-accepted by the participants, yielding a high response rate and a low number of missing values per item (< 1.5%), except for item 4 (“I know that each of my prescribed medications do”) with a higher missingness rate of 9.2%. This higher proportion aligns with findings in the literature: generally, item missingness is very low and below 2.5% (e.g., 20, 32, 36, 37), however, Hellström et al. [31] reported a 6.0% missing value rate for item 4, while Zill et al. [19] observed a percentage as high as 14.8%. The increased number of missing values for this specific item can be attributed to the absence of the category “not applicable” in the German version of the PAM-13 used for the current study (see [105] and **Supplement 1**), along with the fact that not all patients are prescribed medications. Additionally, there is no differentiation between a missing answer and “not applicable” with respect to the proposed analysis strategy [44], even if this answer option is available in the PAM-questionnaire.

Concerning the use of the different item response levels, we observed that the category “strongly disagree” was rarely selected, particularly for the first six items (< 2.5% in each item). Only for item 7, 8 and 9 this category constituted more than 10% of the responses. The limited selection of the “strongly disagree” category also resulted in a disordered threshold for item 1, necessitating a combination of the two levels “strongly disagree” and “disagree” for a good fit of the partial credit model. Furthermore, while we encountered no floor effects, ceiling effects were found for all items except item 9 (“I am confident I can figure out solutions when new problems arise with my health”) and item 11 (“I know how to prevent problems with my health”). The scarce use of the “strongly disagree” category has also been noted in other PAM-13 studies [19, 21, 23, 31, 41], and the presence of ceiling effects is a widespread phenomenon (e.g., 18, 20, 23, 36, 79, 104, 106). It is noteworthy, however, that some studies did not observe any item ceiling effects [24, 26, 79, 107], which might be attributed to the specific cultural background of the population, the age groups considered or the type of disease under investigation.

Aligned with the ceiling effects, the mean overall PAM-13 score in our study was high (69.7, SD = 14.2), placing it into the highest activation level 4 according to the cut-off thresholds (refer to Sect. 2.2.1). This observed average activation surpasses that reported in the validation study conducted by the original PAM-13 developers (mean = 61.9), who examined a sample of individuals aged 45 and older from the general US population (thereof 79% with a chronic disease). Also, in numerous other international validation studies exploring diverse patient populations with varying ages and races, including individuals with osteoarthritis [25], diabetes/hypertension [40, 107], metabolic syndrome [28], cardiac conditions [22], mental health disorders [20] or rheumatic diseases [106], the PAM-13 overall mean scores were lower, ranging between 50.0 [22] and 60.1 [40], and thus classifying into activation level 3 (see also Table 6). Nonetheless, our overall PAM-13 score is well in line with values found in the German validation studies by Brenk-Franz, Hibbard [18] (mean = 68.3, SD = 14.8) and Zill, Dwinger [19] (mean = 67.1, SD not given). An even higher average PAM-score was observed in a healthy Hebrew population without any chronic diseases (mean = 71.9, SD = 15.7) [26]. High overall PAM-13 scores at baseline and item ceiling effects may diminish the PAM-13’s discriminating ability and its responsiveness in capturing changes over time in interventional studies. Extending the PAM-13 at the higher end of the trait continuum with more difficult items to appropriately calibrate the measure for patients with stronger abilities could be beneficial in addressing this issue, as has already been suggested elsewhere [104]. Furthermore, changing the item format of existing items by modifying the question wording or optimizing and extending the response scale may help to reduce ceiling effects [108, 109].

Regarding reliability, the PAM-13 demonstrated a good internal consistency (α = 0.81, ω = 0.84). These results are comparable to previous findings in earlier studies where Cronbach’s α ranged between 0.77 [26] and 0.92 [40, 79], however, our study’s α falls into the lower third of this range, and in the two German PAM-13 validation studies alpha values were higher (α = 0.84 [18] and 0.88 [19]).

Additionally, our study revealed adequate person- and item-reliability. Inter-item and item-rest correlations were mostly moderate to strong. Higher inter-item correlations suggest potential redundancy between items. We found values > 0.50 for items 11 (“I know how to prevent problems with my health”) and 12 (“I am confident I can figure out solutions when new problems arise with my health”). Some other PAM-13 validation studies also identified items which were potentially repetitive with inter-item correlations above 0.5 [27, 36], however, they either did not state the affected items or the affected items differed from ours [36]. Although items 11 and 12 are associated with different activation levels (item 11 = level 3 (“beginning to take action”), item 12 = level 4 (“maintaining behavior over time”)) they both relate to self-management abilities and the prevention of health-related problems. Participants might have implicitly assumed that “knowing how to prevent problems” also requires the confidence to tackle future problems arising with their disease, which might explain the slight redundancy between the two items.

Several studies have established that confidence and self-efficacy are crucial elements of patient activation in disease management [110–112]. In our study, a statistically significant moderate correlation of the PAM-13 with self-efficacy (SES6G) supported the convergent validity of the measure. The correlation with health-related quality of life (EQ-5D) was lower than anticipated (*r* = .22) given the fact that patient activation was shown to be moderately to strongly associated with self-perceived health status and HRQoL in various disease contexts [26, 38, 67, 113, 114]. However, only few of those studies utilized the EQ-5D as a measure of health-related quality of life. We believe that the low correlation may be attributed to the fact not all domains of the EQ-5D can be influenced by patient activation, particularly as mobility and pain/discomfort are heavily dependent on the specific disease status of the individual.

The unidimensionality of the PAM-13 was thoroughly assessed through both confirmatory and exploratory factor analyses. While the confirmatory factor analysis favoured a two-factor model over the one-factor solution, results from the exploratory factor analysis was ambiguous with respect to an underlying one-component structure. Some of the factor selection criteria pointed towards a two-factor solution combining the first three items into “believing active role important and responsibility” (factor 2) and items 4–14 into “knowledge and self-confidence” (factor 1). In the two-factor solution, the explained variance amounted to 48.3%, with the first factor accounting for 37.2% and the second factor for 11.2%. Communalities were low for items 4 and 6, explaining only 16% of the variance in item 4 and 15% of the variance in item 6. Item 4 (“*I know what each of my prescribed medications does*”) is generally problematic due to a higher proportion of missing values, as not all patients are taking medication. By rephrasing the statement to reflect a more general understanding of health-related information rather than focusing specifically on prescribed medication, item 4 could potentially be made more applicable to a broader range of participants. Item 6 (“*I am confident I can tell my health care provider concerns I have, even when he or she does not ask*”) also had the lowest communality in a two-factor model suggested in the study by Bahrom, Ramli [28], who investigated the Malayan version of the PAM-13 among patients with metabolic syndrome. A possible explanation might be that item 6 differs from other items in asking for an external, independent source - the health care provider - whereas all other questions focus on self-managing behaviours and knowledge solely dependent on the specific individual. In the two-factor model, the internal consistency reliability for factor 1 was adequate (α = 0.79), for factor 2 lower with α = 0.69. This value is below the desirable alpha value of > 0.7, however, one must keep in mind that Cronbach’s alpha is impacted by the number of items, and the α value will increase with an increase in number of items. Additionally, some researches have acknowledged the acceptability of lower alphas around 0.6, especially when the item is assessing knowledge or understanding [115]. Total omega values were slightly higher for both factors.

In the context of item response theory, a partial credit model was employed to evaluate the item fit statistics. Both infit and outfit MNSQ indicated a good fit of the model. Using a PCAR resulted in an eigenvalue of < 3 of the first contrast and a variance explained < 15%, and thus providing no evidence conflicting with the assumption of unidimensionality. Furthermore, there was no local response-dependence present, except for potential violations of the independence assumption between item 1 and 2.

In summary, the different analysis techniques applied to assess the factor structure of the PAM-13 within our oncologic patient population did not provide a conclusive picture with respect to dimensionality. This observation is consistent with findings in the existing literature: Some studies did not confirm a unidimensional structure [20, 28, 29, 32, 39, 40, 116], others underscored the ambiguity in their results concerning the latent factors [20, 31]. Notably, authors generally approving the underlying unidimensionality of the PAM-13 with their research, often reported a low proportion of explained variance (< 50%) [19, 21, 22, 79, 107] which might hint towards additional latent factors.

Regarding measurement invariance, we examined potential differential item functioning (DIF) across variables including sex, age, educational level, diagnosis status, and intervention group. Our analysis revealed no discernible evidence of DIF within the assessed parameters. In the literature, results with respect to DIF were inconsistent: Our findings align with Ahn, Yi [25] as well as Moreno-Chico, González-de Paz [30], both of whom similarly reported an absence of relevant evidence for DIF. Contrastingly, most other validation studies observed at least minor DIF for a small number of items with respect to sex [19, 21–23, 41, 79], age [19, 21, 23, 31, 36, 41] and educational level [21–23, 79]. Zill, Dwinger [19] and Ngooi, Packer [22] moreover found DIF for self-rated health status, Lightfoot, Wilkinson [36] and Hung, Carter [41] for disease type.

In line with the identified ceiling effects in item 9 and 11, we observed an item difficulty ranking different from the original order posited by Hibbard, Mahoney [17]. This is a very common result as highlighted above in the comparison between item difficulty rankings in several international PAM-13 validation studies (Sect. 3.5).

Item 9 (“*I know the different medical treatment options available for my health condition”)* emerged as the most challenging item for participants to endorse. Similar observations have been reported in other studies [21, 30, 104], as indicated in Table 6. Moreno-Chico, González-de Paz [30] who investigated the psychometric properties of the PAM-13 in a Spanish population with chronic diseases argued that there might be communication issues in decision-making processes between clinicians and patients contributing to this phenomenon. Graffigna, Barello [21] reasoned that the item order might be influenced by European-specific aspects in the healthcare system, however, as shown in Table 6, item 9 was also harder to endorse by community-dwelling adults in Singapore [104], whereas in another study from Singapore validating the PAM-13 among adults with cardiac conditions, item 9 did not switch to the highest activation level [22]. This finding contradicts the notion that item order can be satisfactorily explained by cultural background. We rather believe that the difficulty of item 9 can be attributed to the complex and very individual treatment regimens in oncology that impede patient-physician communication with respect to all available treatment options.

On the contrary, items 13 (“*I am confident that I can maintain lifestyle changes, like eating right and exercising, even during times of stress*”) and 10 (“*I am able to maintain the lifestyle changes for my health that I have made*”) were regarded as easier. Similar patterns were obtained in previous research: Across all studies outlined in Table 6, when item 10 was perceived as easier, item 13 also dropped in ranking by one to five positions compared to the original order. This observation is not surprising given that both items target the maintenance of health-related behavioral changes. As mentioned by Moreno-Chico, González-de Paz [30], self-efficacy is crucial for the adherence to newly adapted health behaviors [117]. Our study revealed an overall mean self-efficacy (SES6G; range 0–10) score of 7.07 (SD = 1.87) and a statistically significant correlation between patient activation and self-efficacy (*r* = .39). We thus hypothesize that the high level of self-efficacy in our study population may have led to the reordering of items 10 and 13.

Aside from the mistargeting of items mentioned above, there were only minor deviations from the original rankings for the rest of the items. Overall, the correlation between the item order in our CCC-Integrativ study population and the original ranks was higher (*r* = .885) than for most other international validation studies (see Table 6). This is also partially reflected in the relationship between person ability and item difficulty depicted in the person-item map. It is worth noting that, after collapsing the “strongly disagree” and “disagree” categories, the person-item map in our study exhibited reasonably good targeting, and the person abilities did not strongly exceed the difficulty of the items as they have in some other studies [21, 31, 79], where there was a clear lack of items of sufficient difficulty. A mean value of 0.83 (SD = 1.26) for patient location indicates that the sample as a whole was located at a higher item difficulty than the average of the scale, however, mistargeting was not as pronounced as it was observed, e.g., in the study by Hellström, Kassaye Tessma [31] (mean person location = 1.48, SD = 1.66) or Eyles, Ferreira [79]. In our sensitivity analysis employing the GPCM, we found a mean person location of approximately 0 (SD = 0.91). This suggests that relaxing the equality constraint on the item discrimination leads to a good alignment between person abilities and the difficulties of the items on the scale. This alignment was further supported by the test information curve and the standard error plotted against ability levels (see Supplement 5). Consequently, we advocate for the adoption of more complex IRT models in future patient activation research. Nevertheless, it is important to note that some issues inherent to the PAM-13 construct, as elucidated with the PCM analysis above, cannot be fully resolved by applying higher parameter models. This observation is in line with recent work by Holter, Avian [118], who identified the GPCM to be the most suitable model among four different polytomous IRT models (Rating Scale Model, PCM, GPCM, and Graded Response Model) when applied to PAM-13 data collected in an interview setting. Holter and colleagues also encountered a limited use of the “strongly disagree” category, non-sequential item difficulties, poor model fit for several items, and a mismatch between patient abilities and item difficulties, despite applying a GPCM. These findings are thus contributing to a substantial body of patient activation literature and mirror the deficits we observed, which seem to be independent of the choice of IRT model

### Strengths and limitations

A strength of our study is the large sample size encompassing 1125 subjects. This number exceeded the sample size recommended for a valid questionnaire evaluation [119]. Moreover, we followed the high standards of the guidelines by the COSMIN best practice manual [51]. In addition, leveraging the strengths of both CTT and IRT allowed for a comprehensive analysis of the PAM-13 providing a detailed examination of item characteristics and enabling us to compare results with various international PAM-13 validation studies.

Nevertheless, some limitations need to be acknowledged: Firstly, our study is a non-randomized study with outpatients from CCC centres. Prior to the study these individuals were already receiving a comprehensive, multidisciplinary, and specialized care and thus might have exhibited a higher activation compared to other oncology patients in the general population. This applies especially to the participants of the intervention group, as they actively had to approach the counselling centres to participate in the CIH intervention. Yet, despite this potential source for self-selection bias, no differential item functioning was found between the control and intervention group. However, self-selection bias may have partially caused the high item ceiling effects. Secondly, findings are constrained by the fact that the study sample was exclusively drawn from university hospitals in Southern Germany (Baden-Württemberg). Furthermore, the majority of the sample were females (73.0%), primarily diagnosed with mostly mamma carcinoma, which typically affects individuals at a younger age than most other cancer types [120]. Additionally, with 52.5% of individuals holding a higher education entrance qualification or college/university degree, the education level was higher than that of the general German population (32.0% higher education qualification [121]). As such, due to this localized representation and the potential for sex and education bias, generalizability of our results to the broader German population suffering from cancer may be limited.

## Conclusion

Cancer care extends far beyond acute oncologic treatment regimens. Given the trend of a globally ageing population susceptible to oncologic diseases that often require prolonged follow-up and intricate treatment plans due to the chronic and multifaceted nature of the condition, patient activation is crucial in oncology care [7]. Activation empowers affected individuals to navigate the daily challenges of managing the disease and to engage in health-promoting behaviours and shared decision-making with their doctors. Activated patients have been shown to have treatment plans that align better with their preferences and lifestyles [122]. They exhibit better adherence to medication regimens, place a greater focus on preventive care, and are ultimately associated with lower healthcare costs [15]. Thus, an appropriate concept to adequately measure patient activation within the specific population of cancer patients is vital, allowing physicians and healthcare providers to monitor the individuals’ progress and implement tailored interventions based on individual needs to foster activation [123]. Misclassification of patients into incorrect activation levels may result in a flawed understanding of the persons’ abilities and might ultimately lead to potentially mistargeted interventions.

Future research should focus on the revision and refinement of the PAM-13, taking into account the specific diagnostic group, cultural background and the distinctive features of the underlying healthcare system. Strategies may involve modifying existing items, introducing new items, or developing alternative measures that better capture higher levels of patient activation and the specific challenges within the target population. With a valid and reliable measure to adequately quantify patient activation, a better-tailored approach to promoting patient engagement can be provided. This, in turn, has the potential to serve as a pathway to alleviate the burden of cancer care on the healthcare system.

### Electronic supplementary material

Below is the link to the electronic supplementary material.

Supplementary Material 1: Patient Activation Measure 13, German Version (PAM-13-D).

Supplementary Material 2: PAM-13 R-matrix.

Supplementary Material 3: Parallel Analysis Scree Plot.

Supplementary Material 4: Histogram of item ranks for each PAM-13 item across 14 different study populations (see Table 6).

Supplementary Material 5

## Data Availability

The data that support the findings of this study are available from the authors upon reasonable request.

## Abbreviations

(A)GFI: (Adjusted) goodness–of–fit index
CCC: Comprehensive cancer center
CCT: Classical test theory
CFI: Comparative fit index
CIH: Complementary and integrative healthcare
CO: Control group
DIF: Differential item functioning
GPCM: Generalized Partial Credit Model
IG: Intervention group
IRT: Item response theory
KMO: Kaiser–Meyer–Olkin
LID: Local item independence
MAP: Velicer’s minimum average partial
PAM: Patient Activation Measure
PCM: Partial Credit Model
PROM: Patient reported outcome measure
SRMS: Standardized Root Mean Square Residual
TLI: Tucker Lewis Index
VSS: Very simple structure
