# Supplementary material for: The Patient Activation Measure-13 (PAM-13) in an oncology patient population: psychometric properties and dimensionality evaluation
Source: Health Qual Life Outcomes. 2024 May 20;22:39. doi: 10.1186/s12955-024-02255-w (PMC11103863; doi:10.1186/s12955-024-02255-w)
Supplement: Supplementary file 1 — Supplementary Material 1: Patient Activation Measure 13, German Version (PAM-13-D). [file 12955_2024_2255_MOESM1_ESM.docx]

| Item |  | stimme überhaupt nicht zu | | stimme nicht zu | stimme zu | stimme voll und ganz zu |
| --- | --- | --- | --- | --- | --- | --- |
| 1 | Letzten Endes bin ich selbst dafür verantwortlich, mich um meine  Erkrankung zu kümmern. | ❑ | | ❑ | ❑ | ❑ |
| 2 | Um meine Gesundheit und meine Leistungsfähigkeit zu beeinflussen, ist es am wichtigsten, eine aktive Rolle im Rahmen meiner Behandlung einzunehmen. | ❑ | | ❑ | ❑ | ❑ |
| 3 | Ich bin davon überzeugt, dass ich Maßnahmen ergreifen kann, die helfen, Symptome und Probleme meiner Erkrankung zu verhindern oder zu verringern. | ❑ | | ❑ | ❑ | ❑ |
| 4 | Ich weiß, was jedes der mir verschriebenen Medikamente bewirken soll. | ❑ | ❑ | | ❑ | ❑ |
| 5 | Ich bin davon überzeugt, dass ich beurteilen kann, wann ich eine medizinische Behandlung benötige und wann ich ein Gesundheitsproblem selbst bewältigen kann. | ❑ | | ❑ | ❑ | ❑ |
| 6 | Ich bin davon überzeugt, dass ich meinem Behandler meine Anliegen mitteilen kann, auch wenn er nicht danach fragt. | ❑ | | ❑ | ❑ | ❑ |
| 7 | Ich bin davon überzeugt, dass ich medizinische Behandlungen, die ich zu Hause durchführen muss, auch umsetzen kann. | ❑ | | ❑ | ❑ | ❑ |
| 8 | Ich weiß über meine Erkrankung und ihre Ursachen Bescheid. | ❑ | | ❑ | ❑ | ❑ |
| 9 | Ich kenne die verschiedenen Behandlungsmöglichkeiten für meine Erkrankung. | ❑ | | ❑ | ❑ | ❑ |
| 10 | Ich bin in der Lage, die Veränderungen meiner Lebensgewohnheiten aufrechtzuerhalten, die ich aufgrund meiner Erkrankung begonnen habe. | ❑ | | ❑ | ❑ | ❑ |
| 11 | Ich weiß, wie ich weitere Probleme mit meiner Erkrankung verhindern kann. | ❑ | | ❑ | ❑ | ❑ |
| 12 | Ich bin davon überzeugt, dass ich für neu auftretende Situationen oder Probleme mit meiner Erkrankung Lösungen finden kann. | ❑ | | ❑ | ❑ | ❑ |
| 13 | Ich bin davon überzeugt, dass ich Veränderungen meiner Lebensgewohnheiten, wie eine gesunde Ernährung und Sport, auch in stressigen Zeiten beibehalten kann. | ❑ | | ❑ | ❑ | ❑ |

**Supplement 1: Patient Activation Measure 13, German Version (PAM-13-D)**
