# Supplementary figures and images for: The Patient Activation Measure-13 (PAM-13) in an oncology patient population: psychometric properties and dimensionality evaluation
Source: Health Qual Life Outcomes. 2024 May 20;22:39. doi: 10.1186/s12955-024-02255-w (PMC11103863; doi:10.1186/s12955-024-02255-w)

**Supplement 2:** PAM-13 R-matrix


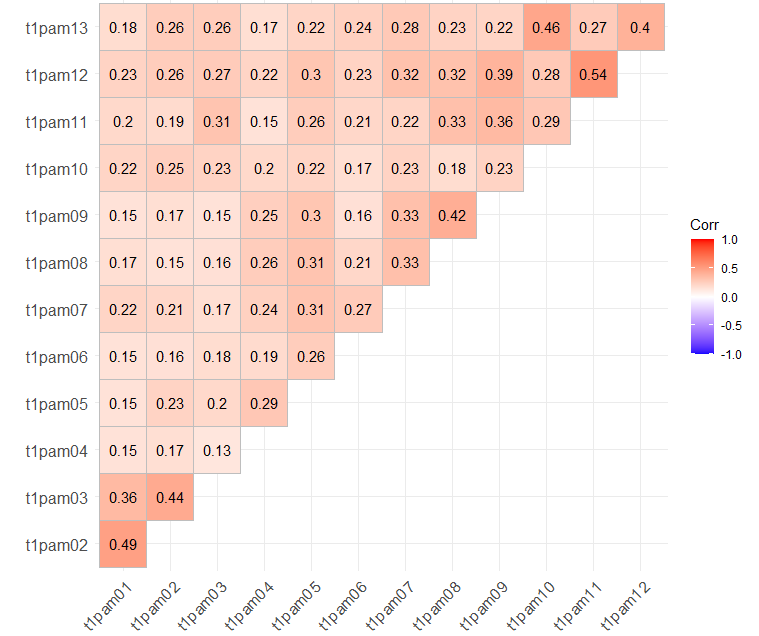

Supplement: Supplementary file 2 — Supplementary Material 2: PAM-13 R-matrix. [file 12955_2024_2255_MOESM2_ESM.docx]

**Supplement 3:** Parallel Analysis Scree Plot

**
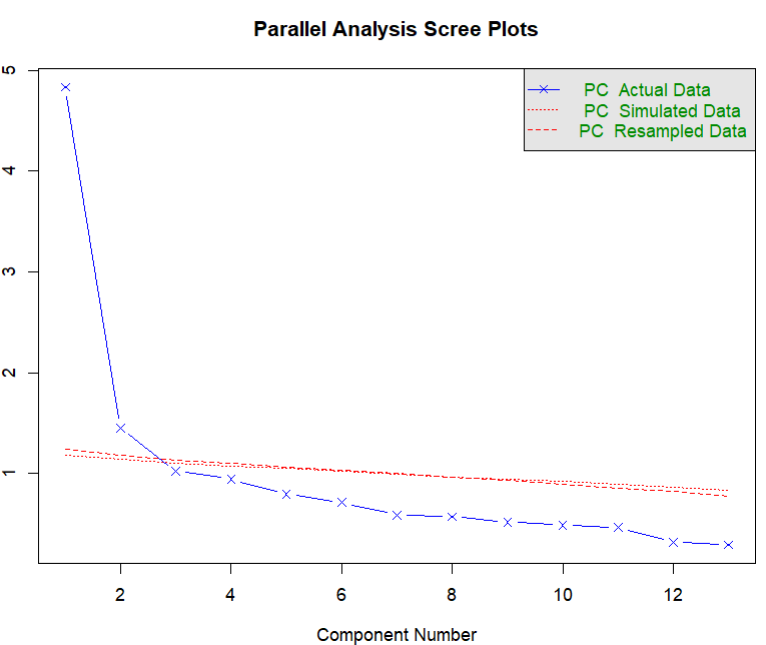
**

Supplement: Supplementary file 3 — Supplementary Material 3: Parallel Analysis Scree Plot. [file 12955_2024_2255_MOESM3_ESM.docx]
