# Supplementary material for: The Patient Activation Measure-13 (PAM-13) in an oncology patient population: psychometric properties and dimensionality evaluation
Source: Health Qual Life Outcomes. 2024 May 20;22:39. doi: 10.1186/s12955-024-02255-w (PMC11103863; doi:10.1186/s12955-024-02255-w)
Supplement: Supplementary file 4 — Supplementary Material 4: Histogram of item ranks for each PAM-13 item across 14 different study populations (see Table 6). [file 12955_2024_2255_MOESM4_ESM.docx]

**Supplement 4:** Histogram of item ranks for each PAM-13 item across 14 different study populations (see Table 6)

| 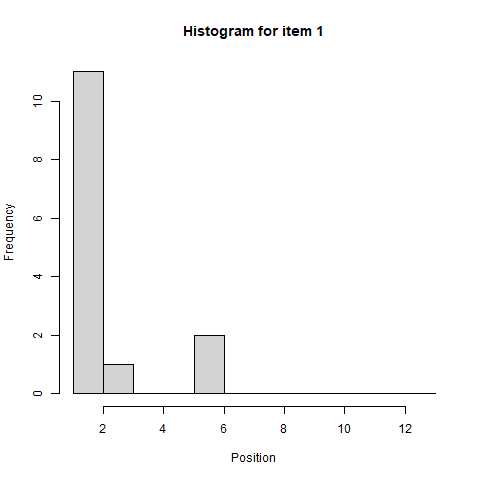 | 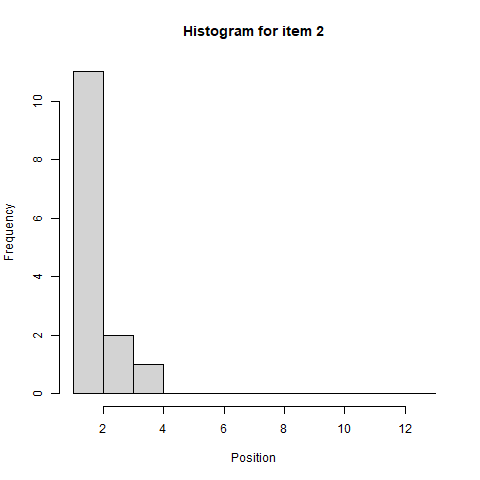 |
| --- | --- |
| 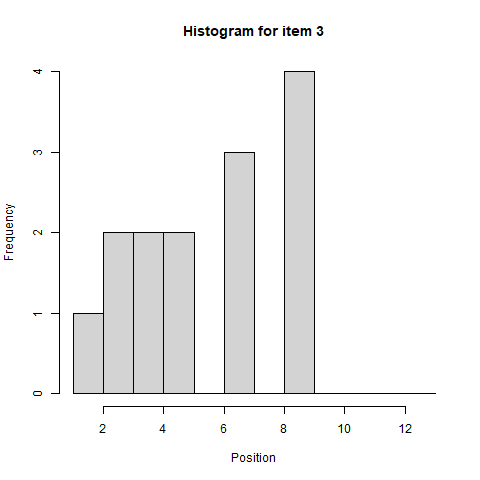 | 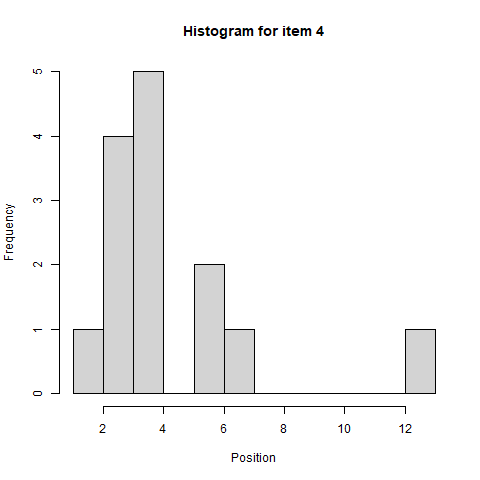 |
| 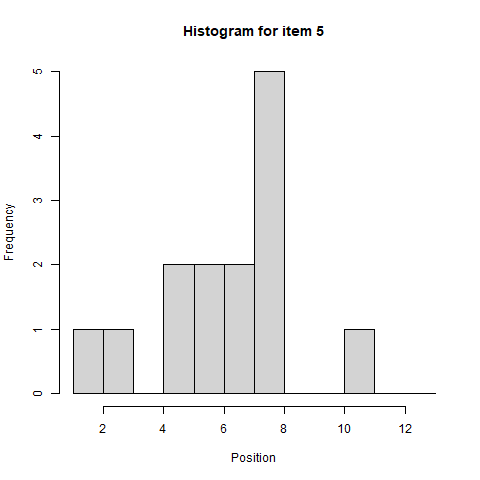 | 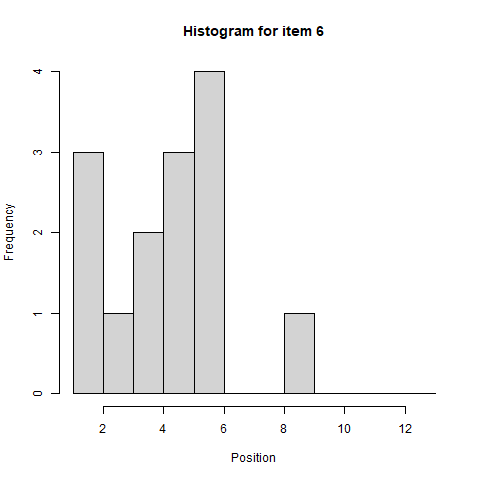 |
| 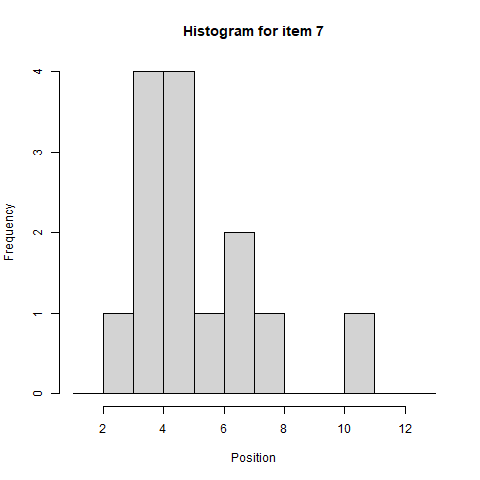 | 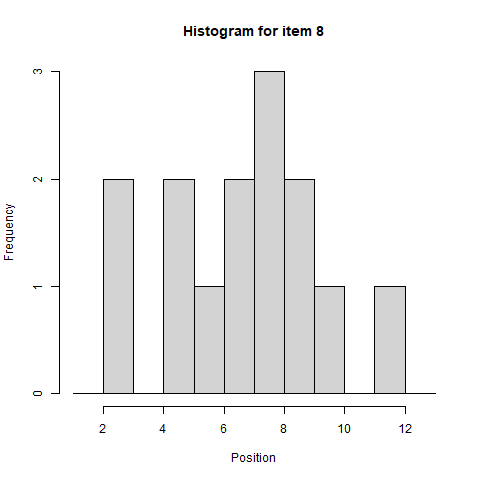 |
| 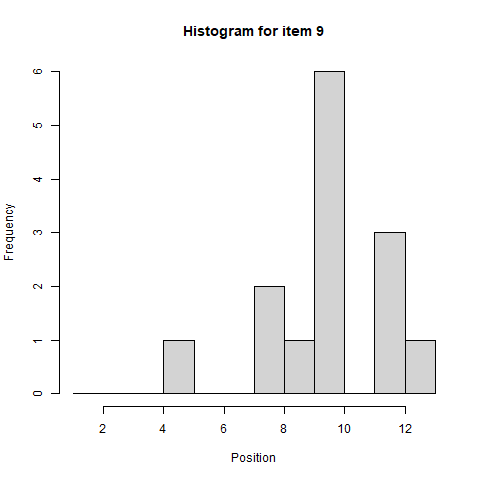 | 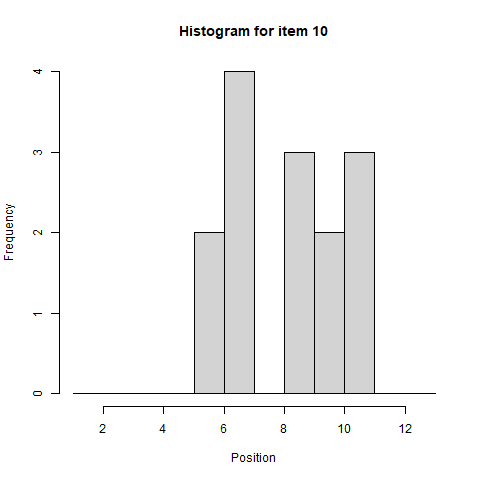 |
| 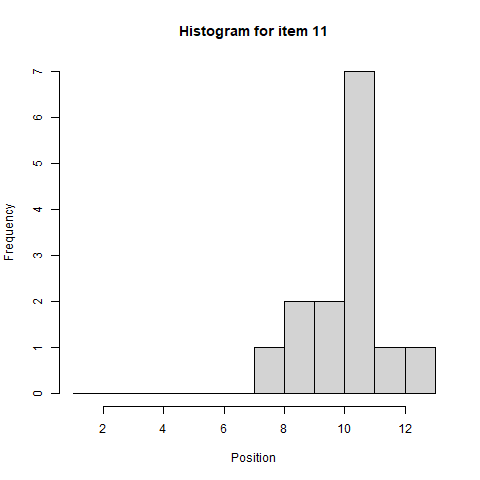 | 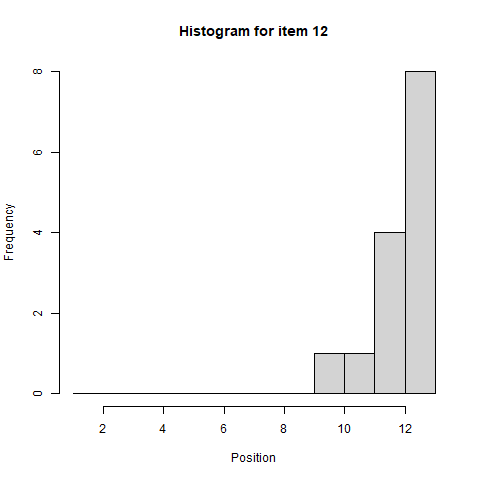 |
| 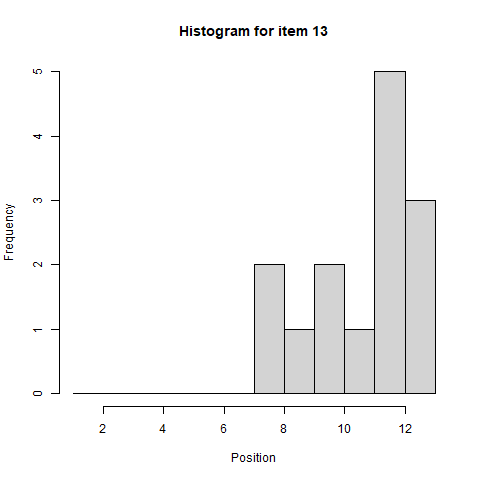 |  |
