# Supplementary material for: The Patient Activation Measure-13 (PAM-13) in an oncology patient population: psychometric properties and dimensionality evaluation
Source: Health Qual Life Outcomes. 2024 May 20;22:39. doi: 10.1186/s12955-024-02255-w (PMC11103863; doi:10.1186/s12955-024-02255-w)

**Supplement 5:** Sensitivity Analysis: Generalized partial credit model (GPCM)

**Supplementary Table 5.1:** Model diagnostics: PCM vs. GPCM

|  | *AIC* | *SABIC* | *TLI* | *CFI* | *RMSEA* | *95% CI of RMSEA* |
| --- | --- | --- | --- | --- | --- | --- |
| **PCM** | 25278.57 | 25328.50 | 0.89 | 0.91 | 0.089 | 0.082; 0.096 |
| **GPCM** | 25244.12 | 25316.24 | 0.92 | 0.93 | 0.076 | 0.070; 0.835 |

AIC = Akaike information criterion, SABIC=sample adjusted Bayesian information criterion, TLI=Tucker-Lewis index, CFI=comparative fit index,

RMSEA=root mean square error of approximation, CI = confidence interval.

**Supplementary Table 5.2:** Item fit statistics of the GPCM model

| *Item order PCM* | *Item order GPCM* | *Difficulty* | *alpha* | *Threshold 1* | *Threshold 2* | *Outfit MNSQ* | *Outfit z* | *Infit*  *MNSQ* | *Infit z* |
| --- | --- | --- | --- | --- | --- | --- | --- | --- | --- |
| 1 | 4 | -1.784 | 0.808 | -2.405 | -1.162 | 0.917 | -1.356 | 0.955 | -0.919 |
| 2 | 1 | -1.743 | 1.129 | -2.737 | -0.749 | 0.940 | -0.939 | 0.923 | -1.670 |
| 4 | 2 | -1.560 | 1.172 | -2.708 | -0.412 | 0.887 | -2.140 | 0.910 | -2.162 |
| 3 | 3 | -1.207 | 1.068 | -2.357 | -0.057 | 0.914 | -1.908 | 0.925 | -1.858 |
| 6 | 6 | -0.745 | 0.787 | -1.88 | 0.389 | 0.927 | -1.902 | 0.945 | -1.456 |
| 5 | 5 | -0.521 | 1.107 | -1.804 | 0.762 | 0.872 | -3.161 | 0.886 | -2.804 |
| 10 | 10 | -0.464 | 0.993 | -1.553 | 0.625 | 0.883 | -3.017 | 0.898 | -2.678 |
| 7 | 7 | -0.106 | 0.901 | -0.647 | 0.435 | 0.883 | -3.102 | 0.903 | -2.809 |
| 8 | 8 | 0.168 | 0.967 | -0.692 | 1.029 | 0.899 | -2.664 | 0.915 | -2.329 |
| 13 | 13 | 0.246 | 1.079 | -0.934 | 1.425 | 0.912 | -2.255 | 0.918 | -2.080 |
| 12 | 12 | 0.453 | 1.595 | -0.402 | 1.309 | 0.797 | -4.762 | 0.819 | -4.639 |
| 11 | 11 | 0.524 | 1.325 | -0.497 | 1.544 | 0.873 | -3.156 | 0.868 | -3.374 |
| 9 | 9 | 0.783 | 1.055 | -0.148 | 1.713 | 0.877 | -3.004 | 0.898 | -2.612 |

From lighter to darker: (i) Believes active role is important; (ii) Confidence and knowledge to take action; (iii) Taking action; (iv) Staying the course under stress.

**Supplementary Figure 5.1:** Person-item-map


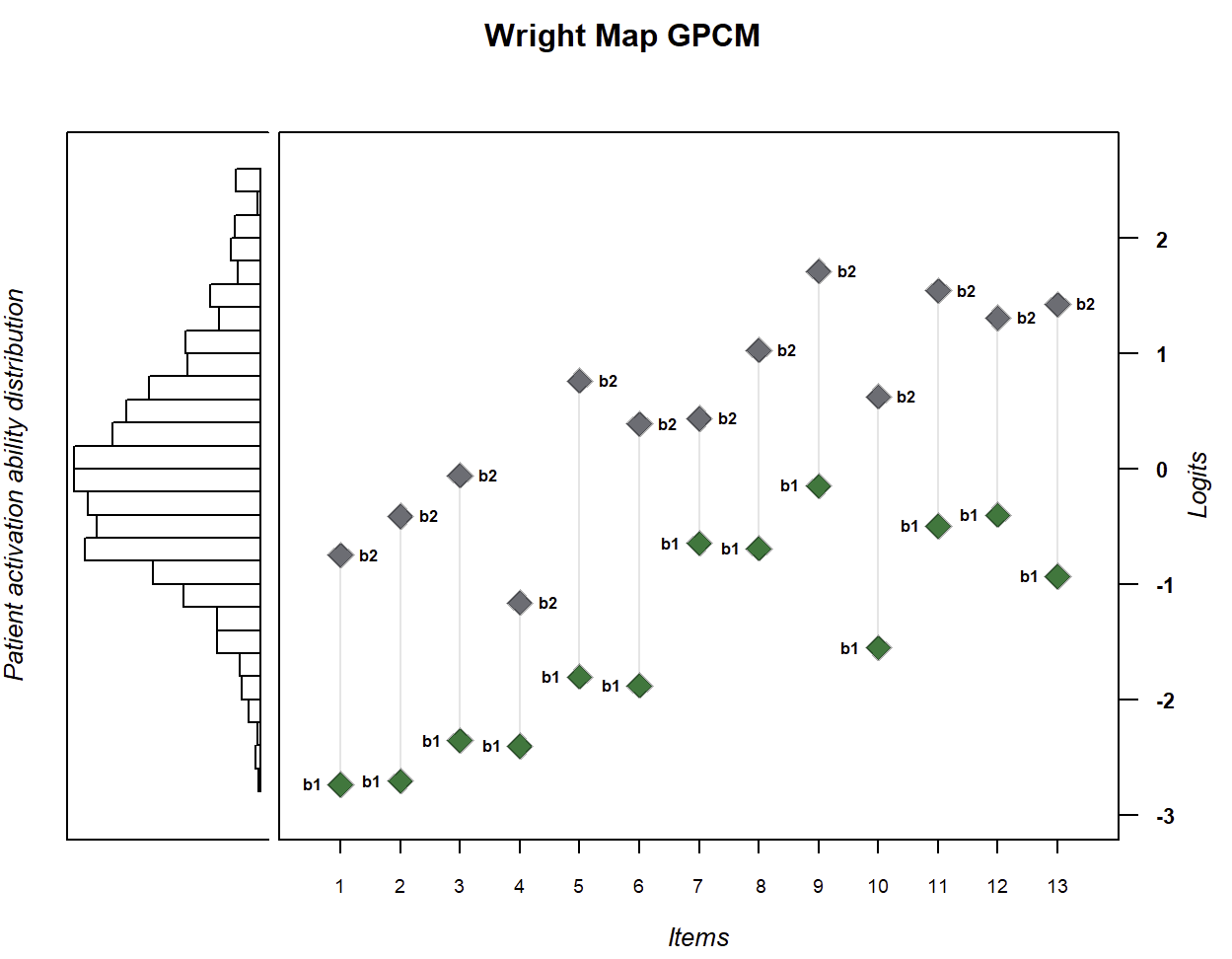


**Supplementary Figure 5.2:** Test information (green) and standard error (grey)


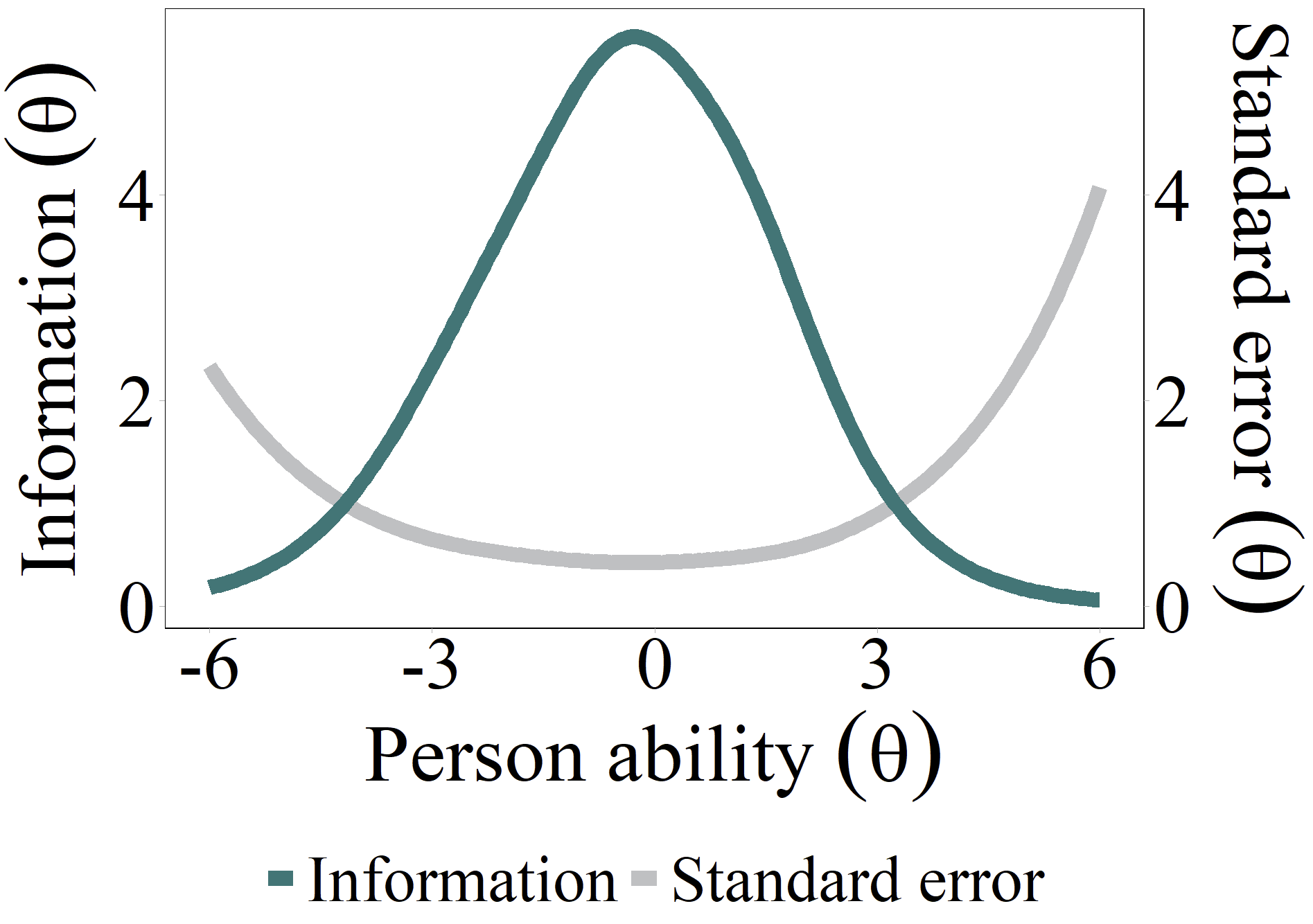

Supplement: Supplementary file 5 — Supplementary Material 5 [file 12955_2024_2255_MOESM5_ESM.docx]
